# Genetic and antimicrobial resistance profiles of non-O157 Shiga toxin-producing *Escherichia coli* from different sources in Egypt

**DOI:** 10.1186/s12866-021-02308-w

**Published:** 2021-09-23

**Authors:** Mohamed Sabry Abd Elraheam Elsayed, Samah Mahmoud Eldsouky, Tamer Roshdy, Abeer Mohamed Ahmed Bayoume, Ghada M. Nasr, Ali S. A. Salama, Behiry A. Akl, Al Shaimaa Hasan, Amany Kasem Shahat, Rana Atef Khashaba, Walid Abdellatif Abdelhalim, Hend E. Nasr, Lina Abdelhady Mohammed, Ahmed Salah

**Affiliations:** 1 Department of Bacteriology, Mycology, and Immunology, Faculty of Veterinary Medicine, University of Sadat City, Sadat City, Menoufia Egypt; 2 Department of Otolaryngology and Head and Neck Surgery, Faculty of Medicine, Benha University, Benha, Egypt; 3 Department of Molecular Biology, Genetic Engineering and Biotechnology Research Institute, University of Sadat City, Sadat City, Menoufia Egypt; 4 Department of Microbial Biotechnology, Genetic Engineering and Biotechnology Research Institute, University of Sadat City, Sadat City, Menoufia Egypt; 5 Department of Molecular Diagnostics, Genetic Engineering and Biotechnology Research Institute, University of Sadat City, Sadat City, Menoufia 32897 Egypt; 6 Microbiology Department, Faculty of Agriculture, Zagazig University, Zagazig, Egypt; 7 Department of Medical Pharmacology, Qena Faculty of Medicine, South Valley University, Qena, Egypt; 8 Department of Medical Microbiology and Immunology, Benha University, Benha, Egypt; 9 Department of Clinical Pathology and Chemistry, Benha Faculty of Medicine, Benha University, Benha, Egypt; 10 Department of Medical Biochemistry and Molecular Biology, Benha University, Benha, Egypt

**Keywords:** Shiga toxin-producing *Escherichia coli*, Animals and human, Virulence genes, Antimicrobial resistance genes, MLVA genotyping

## Abstract

**Background:**

The Shiga toxin-producing *Escherichia coli* (STEC) represented a great risk to public health. In this study, 60 STEC strains recovered from broiler and duck fecal samples, cow’s milk, cattle beef, human urine, and ear discharge were screened for 12 virulence genes, phenotypic and genotypic antimicrobial resistance, and multiple-locus variable-number tandem-repeat analysis (MLVA).

**Results:**

The majority of strains harbored Shiga toxin 1 (*stx*_1_) and *stx*_1d_, *stx*_2_ and *stx*_2e_, and *ehx*A genes, while a minority harbored *stx*_2c_ subtype and *eae*A. We identified 10 *stx* gene combinations; most of strains 31/60 (51.7%) exhibited four copies of *stx* genes, namely the *stx*_1_, *stx*_1d_, *stx*_2_, and *stx*_2e_, and the strains exhibited a high range of multiple antimicrobial resistance indices. The resistance genes *bla*CTX-M-1 and *bla*TEM were detected. For the oxytetracycline resistance genes, most of strains contained *tet*A, *tet*B, *tet*E, and *tet*G while the *tet*C was present at low frequency. MLVA genotyping resolved 26 unique genotypes; genotype 21 was highly prevalent. The six highly discriminatory loci DI = 0.9138 are suitable for the preliminary genotyping of STEC from animals and humans.

**Conclusions:**

The STEC isolated from animals are virulent, resistant to antimicrobials, and genetically diverse, thus demands greater attention for the potential risk to human.

**Supplementary Information:**

The online version contains supplementary material available at 10.1186/s12866-021-02308-w.

## Background

Shiga toxin-producing *Escherichia coli* (STEC) is a pathotype of *E. coli* that produces Shiga toxins which show molecular similarity to that produced by *Shigella dysenteriae* type 1 [1]. The STEC serotypes are major foodborne pathogens that cause non-bloody to bloody diarrhea that may ultimately lead to hemolytic uremic syndrome (HUS) [2]. Although ruminants are the main reservoir of STEC that contaminate environment and foods of animal and plant origins, STEC isolates were confirmed in poultry, psittacine pet birds, wild birds and pigeons, dogs, and pigs [3]. Many human outbreaks were caused by the top six STEC serogroups, notably O26, O45, O103, O111, O121, and O145 [4]. The incidence of infections caused by one of the top six STEC serogroups in the USA soared from 0.19 per 100.000 in the year 2007 to 0.79 per 100.000 in the year 2014 [1]. The lipopolysaccharide antigen enables *E. coli* to colonize bladders and increases bacterial resistance against hydrophobic antibiotics [5, 6].

Shiga toxins 1 and 2 are major virulence factors of STEC. There are three subtypes of the *stx*_1_ gene, including *stx*_1a_, *stx*_1c_, and *stx*_1d_ and ten subtypes of *stx*_2_, indicated as *stx*_2a-2k_ [7–10]. Globotriaosylceramide receptors in eukaryotic cell membranes are the binding targets for the Stx1B subunits and for the B subunits of most Stx2 types, whereas; the globotetraosylceramide is the target for Stx2e B subunits [11]. The DNA sequence encoding Stx1 is highly conserved and only a few Stx1 subtypes have been reported; by contrast, Stx2 has numerous subtypes within a range of 84–99% sequence similarity [12, 13]. The Stx2 toxin has been associated with severe disease [14]; it is a 1000-times more toxic than Stx1 to renal microvascular endothelial cells, and *stx*_2c_ subtype is more commonly reported in patients suffering from HUS [15]. Fecal samples from healthy humans and animals, as well as feces-contaminated environments and foods contain a high burden of STEC strains and phages carrying the *stx* gene [16]; these can lysogenize non-virulent bacterial strains and convert them into Shiga toxin-producing types [17]. Intimin is a protein encoded by the bacterial *eae* gene. It is a component of the enterocyte effacement complex which generates attaching and effacing lesions. The majority of STEC infections that produce serious complications like hemolytic uremic syndrome (HUS) are caused by bacteria that attach to and efface enterocytes (LEE). However, HUS has been linked to a subset of STEC isolates that do not possess LEE [18]. Likewise, enterohemolysin (*ehx*A) disrupts the cytoplasmic membranes of mammalian cells [14]; intimin and enterohemolysin contribute to STEC-associated virulence.

The development and transmission of antimicrobial-resistant STEC have become tremendous problems worldwide, as these bacteria may be transferred from poultry to the human food chain [19], and from cattle through farm manure runoff and occupational exposure [20]. Continuous monitoring of antimicrobial-resistant STEC provides information on the development of resistant serotypes, the dynamics of bacterial transmission, and antimicrobial abuse [20]. Furthermore, extended-spectrum β-lactamase (ESBL) encoded by the *bla*CTX-M gene has been identified on plasmids; most hospital- and community-acquired infections with *E. coli* or *Klebsiella pneumoniae* are associated with strains that contain *CTX*-M-type genes [21]. More than 30 different genes encoding for resistance to tetracycline have been identified, involving two major resistance mechanisms: those that promote active efflux of antibiotics and others that prevent interactions with the bacterial ribosomes. The genes *tet* (A, B, C, D, E, and G) encode control proteins that promote active efflux [22].

Multiple-locus variable-number tandem-repeat (MLVA) analysis has recently emerged as a rapid and highly discriminatory technique for *E. coli* genotyping [23–25]. The methodology detects polymorphisms within variable number tandem repeats (VNTRs) that are present in loci dispersed over the bacterial genome. Some of these VNTRs display critical polymorphisms that can distinguish between highly related clonal strains. MLVA is a promising tool for bacterial genotyping and may even be more effective than pulsed-field gel electrophoresis which is currently the gold standard for *E. coli* genotyping [23, 26]. MLVA was successfully implemented for elucidating the molecular epidemiology of the *E. coli* O157:H7 strain [27]. Izumiya et al. [28] confirmed the applicability of this method for genotyping of O26 and O111. Researchers can perform efficient STEC subtyping using MLVA, which is a very sensitive tool. Future epidemiological investigations of STEC clonality, including both O157 and non-O157 isolates, could benefit from the diversity present in many serotypes [29].

The purposes of this study were to determine the prevalence of STEC strains among different animal and human samples. And to detect various Shiga toxin genes and their subtypes, phenotypic antimicrobial resistance, extended-spectrum β-lactamases, and tetracycline resistance genes in STEC from diverse sources in a large geographic area in Egypt. To unravel the associations between phenotypic and genotypic features and origin of strains. Moreover, to compare the MLVA profiles with Shiga toxin gene combinations to understand any genetic similarities between animal and human strains. To evaluate the discriminatory power of MLVA loci combinations for elucidating the vital MLVA combination that could be used in preliminary molecular epidemiological studies when rapid results are required.

## Materials and methods

### Sampling, isolation, and identification

During the year 2018, a total of 207 samples were collected, including 15 and 20 fecal swabs from broilers and ducks, respectively, 10 samples of cattle meat, 12 samples of cow’s milk, 50 samples of human urine from cases with urinary tract infections, and 100 swabs of human ear discharge from cases with otitis media, the urine samples and ear swabs were collected from separate human cases. For the fecal and meat samples 25 g were collected, while for milk samples 10 ml were collected after discarding the first strips, and 10 ml urine samples were collected from the midstream urine. The human cases are cattle farm workers and farmers rearing broilers and ducks on the small scale. The chicken, duck, and cow’s milk samples were collected from farms at Sadat City, Minoufia, Egypt, and these samples were collected after a written informed consent from the owner. This research was performed according to the recommendations of the U.S. Government for the utilization and care of vertebrate animals used in testing, research, and training. The cattle meat samples were collected from 10 butcher shops at Sadat City, and the urine and ear discharge samples were collected from the Central Hospital of Sadat City and Benha University Hospital, respectively. An Informed consent was obtained from all human participants. The adopted methods for handling of human samples were carried out in accordance with relevant guidelines and regulations provided in the Declaration of Helsinki. The fecal, meat, and ear discharge samples were collected in sterile plastic bags and the milk and urine samples were collected in 10 ml sterile tubes. All the samples were labeled, packed, cooled in an icebox, and transported immediately to the Central Bacteriology, Mycology, and Immunology laboratory at the Faculty of Veterinary Medicine, University of Sadat City and stored at 4 °C prior to analysis; all samples were processed as fast as possible to optimize the quality of the findings. The isolation and identification procedures of STEC strains comply with the methods outlined by the U. S. Food and Drug Administration Bacteriological Analytical Manual (FDA-BAM) [30]. Briefly, the samples were used to inoculate 225 ml of brain heart infusion broth which was incubated at 35 °C for 3 h to promote resuscitation of potentially injured cells. These pre-enriched samples were then transferred to 225 ml of tryptone phosphate broth and incubated at 37 °C for 20 h. One ml of the enriched broth was plated onto Levine’s eosin-methylene blue (EMB) and MacConkey (MAC) agar plates which were incubated for 18-24 h at 35 °C. Colonies with the characteristic metallic sheen on EMB agar were selected for STEC identification and analysis using biochemical tests. The presumptive STEC colonies (3–5) were cultured onto citrate utilization (−), triple sugar iron (− H_2_S), and urea agar slants (−ve), and were also subjected to the catalase (+), methyl-red (+), indole (+), and Voges–Proskauer (−) tests; ability of fermenting glucose and lactose sugars (+) was also evaluated. The isolates that exhibited the anticipated results were recorded as STEC isolates [31].

The STEC isolates were subjected to further confirmation using Biolog GN2 and GP2 MicroPlates (Gram-negative and Gram-positive tests, respectively) and the Biolog Microlog 3.70 database and software (Biolog, Hayward, Calif.) [32]. *E. coli* American type culture collection (ATCC) 11775 and *Salmonella enterica* subsp. enterica Berta ATCC 8392 were included as positive and negative controls, respectively.

The study design and all the experimental protocols were approved by the Committee for Animal Care and Use, Faculty of Veterinary Medicine, University of Sadat City, Egypt, and the given number was 2018–50. In addition, the committee approved the utilization of human samples within this study after the revision of the informed consent form.

### Detection of O-serogroups

The confirmed isolates were re-cultivated on MAC agar and submitted to the Central Laboratories of Ministry of Health, Egypt. Slide agglutination tests were performed using commercial monovalent and polyvalent antisera supplied by Denka-Seiken (Japan).

### Antimicrobial susceptibility patterns of STEC strains

The antimicrobial susceptibility patterns of STEC strains were detected after screening the isolates against a panel of 13 antimicrobial agents (Oxoid, UK; Table 1), selected on the basis of their medical importance. One ml of the calibrated bacterial suspension (0.5 McFarland standard units) which include 1.5 × 10^8^ colony forming unites/ ml were inoculated on Mueller-Hinton agar; *E. coli* ATCC 25922 was utilized as the quality control. The Kirby-Bauer disk diffusion method was utilized to determine antibiotic sensitivity; the results were interpreted according to Clinical Laboratory and Standards Institute criteria [33]. The multiple antimicrobial resistance index (MAR) for each strain was determined by dividing the number of antimicrobials to which the strain was resistant by the total number of antimicrobials used.

**Table 1:** Types, groups, and prioritization of antimicrobials classified as critically important in human and veterinary medicine

Antimicrobial agents	Disk concentration	Antimicrobial class	Medical importance	Prioritization criterion
Amikacin	30 μg	Aminoglycosides	High priority critically important	P2 and P3
Amoxicillin/ clavulanic acid	30 μg	Penicillins	Highest priority critically important	P2 and P3
Ampicillin	10 μg	Penicillins	Highest priority critically important	P2 and P3
Cephradine	10 μg	Cephalosporins	Highly important	NA
Chloramphenicol	15 μg	Amphenicols	Highly important	NA
Clindamycin	20 μg	Lincosamides	Highly important	NA
Doxycycline	20 μg	Tetracyclines	Highly important	NA
Erythromycin	20 μg	Macrolides and ketolides	Highest priority critically important	P1, P2 and P3
Nalidixic acid	30 μg	Quinolones	Highest priority critically important	P1, P2 and P3
Norocillin	10 μg	Penicillins	High priority critically important	P2 and P3
Oxytetracyclin	20 μg	Tetracyclines	Highly important	NA
Penicillin G	10 μg	Penicillins	High priority critically important	P2 and P3
Streptomycin	5 μg	Aminoglycosides	High priority critically important	P2 and P3

**Prioritization criterion 1 (P1)**: an antimicrobial used widely among patients with critical infections and in bacterial diseases in health care settings for which this antimicrobial class is the only or one of few alternatives available. **Prioritization criterion 2 (P2)**: an antimicrobial used widely and of the class that may be useful for treating critical infections in health care settings but whose use may favor the generation of resistance. **Prioritization criterion 3 (P3)**: The antimicrobial class typically chosen to control infections in those infected with resistant bacteria or bacteria that harbor resistance genes from non-human origins; NA, no prioritization has been assigned.

### Molecular typing techniques

Genomic DNA was extracted using the QIAamp kit (Qiagen, Hilden, Germany) as per the manufacturer’s instructions. All isolates were screened for virulence genes including *stx*_1_, *stx*_1c_, *stx*_1d_, *stx*_2_, *stx*_2a_, *stx*_2c_, *stx*_2d_, *stx*_2e_, *stx*_2f_, *stx*_2g_, *eae*A, and *ehx*A. These isolates were also screened for class 1 and 2 integrons (*int*l1 and *int*l2), extended-spectrum β-lactamase (*bla*CTX-M and *bla*CTX-M-1), and the ampicillin-resistance gene (*bla*TEM; Table 2). Screening for oxytetracycline resistance genes *tet*A, *tet*B, *tet*C, *tet*D, *tet*E, and *tet*G was performed. The PCR protocol for the virulence and resistance genes using primers listed in (Table 5) was as follows; the 25 μl reaction volume contained 12.5 μl of ready-to-use master mix, 2 μl of bacterial genomic DNA (100 ng/μl), 0.5 μl of each upstream and downstream primer (50 pmol/μl), and 9.5 μl of RNase-free water. The efficiency of PCR amplification for detecting Shiga toxin and antimicrobial resistance genes was observed by the integration of internal positive control from the tested STEC isolates. Genotyping with eight MLVA loci was performed and amplification of the VNTR target loci was modified to be a single reaction with a final volume of 10 μl that included 1 μl of 10X PCR Mg^2+^ free buffer (Invitrogen, Carlsbad, CA, USA), 2 μM MgCl_2_, 1 U of Platinum *Taq* polymerase (Invitrogen), 0.2 mM of PCR Nucleotide Mix (Roche Applied Sciences), 1 μl of DNA template, and primers at concentrations of 0.6, 0.2, 0.12, 0.36, 0.6, 0.02, 0.012, and 0.03 μm to amplify VNTR3, 9, 25, 34, 17, 19, 36, and 37, respectively. Primers and reagents were supplied by Takara Holdings, Japan [39].

**Table 2:** Primer sequences, anticipated amplicon size, and amplification conditions

Name	Amplicon size (bp)	Cycle Number	Annealing Temperature (°C) and Time	Primer sequence	Purpose	Reference
*stx*_1_	348	25	56 °C, 60s	F: 5′-CAGTTAATGTGGTGGCGAAGG-3′ R: 5′-CACCAGACAATGTAACCGCTG-3’	Detect Shiga toxins and their subtypes [14]	[14]
*stx*_1c_	498	30	56 °C, 60s	F:TTTTCACATGTTACCTTTCCT R:CATAGAAGGAAACTCATTAGG		[34]
*stx*_1d_	192	30	56 °C, 60s	F:CTTTTCAGTTAATGCGATTGCT R:AACCCCATGATATCGACTGC		
*stx*_2_	584	25	56 °C, 60s	F:ATCCTATTCCCGGGAGTTTACG R:GCGTCATCGTATACACAGGAGC		[14]
*stx*_2a_	349	25	65 °C, 40s	F:GCGATACTGRGBACTGTGGCC R:CCGKCAACCTTCACTGTAAATGTG		[35]
*stx*_2c_	124	30	65 °C, 40s	F:GCGGTTTTATTTGCATTAGT R:AGTACTCTTTTCCGGCCACT		[34]
*stx*_2d_	175	30	65 °C, 40s	F:GGTAAAATTGAGTTCTCTAAGTAT R:CAGCAAATCCTGAACCTGACG		
*stx*_2e_	267	30	65 °C, 40s	F:ATGAAGAAGATGTTTATAGCG R:TCAGTTAAACTTCACCTGGGC		
*stx*_2f_	428	30	65 °C, 40s	F:AGATTGGGCGTCATTCACTGGTTG R:TACTTTAATGGCCGCCCTGTCTCC		
*stx*_2g_	573	30	65 °C, 40s	F:GTTATATTTCTGTGGATATC R:GAATAACCGCTACAGTA		
*eae*	682	35	58 °C, 20s	F:ATTACTGAGATTAAGGCTGAT R:ATTTATTTGCAGCCCCCCAT	Detect intimin [14]	[36]
*ehx*A	166	25	56 °C, 60s	F:GTTTATTCTGGGGCAGGCTC R:CTTCACGTCACCATACATAT	Detect enterohemo-lysin [14]	[14]
*tet*A	210	30	58 °C, 60s	F:GCTACATCCTGCTTGCCTTC R:CATAGATCGCCGTGAAGAG	Detect *Tet* (A, B, C, D, E, and G), control active efflux of tetracyclin [22]	[37]
*tet*B	659	30	56 °C, 60s	F:TTGGTTAGGGGCAAGTTTTG R:GTAATGGGCCAATAACACCG		
*tet*C	418	30	58 °C, 60s	F:CTTGAGAGCCTTCAACCCAG R:ATGGTCGTCATCTACCTGCC		
*tet*D	787	30	60 °C, 60s	F:AAACCATTACGGCATTCTGC R:GACCGGATACACCATCCATC		
*tet*E	278	30	58 °C, 60s	F:AAACCACATCCTCCATACGC R:AAATAGGCCACAACCGTCAG		
*tet*G	468	30	60 °C, 60s	F:GCTCGGTGGTATCTCTGCTC R:AGCAACAGAATCGGGAACAC		
*int*l1	280	33	64 °C, 30s	F: CCTCCCGCACGATGATC R: TCCACGCATCGTCAGGC	Detect class 1 and 2 integrons [38]	[21]
*int*I2	300	33	64 °C, 30s	F: GCAAACGCAAGCATTCATTA R: ACGGATATGCGACAAAAAGG		
*bla*CTX-M	500	35	55 °C, 1 min	F: TTTGCGATGTGCAGTACCAGTAA R: CTCCGCTGCCGGTTTTATC	Detect extended-spectrum β-lactamase *(E. coli*) [38]	
*bla*CTX-M-1	415	35	55 °C, 1 min	F: AAAAATCACTGCGCCAGTTC R: AGCTTATTCATCGCCACGTT		
*bla*TEM	800	30	55 °C, 1 min	T1: CCGTGTCGCCCTTATTCC T2: AGGCACCTATCTCAGCGA	Detect ampicillin-resistance gene [38]	
VNTR-3	(333–334) to (476–477)	35	65 °C, 20s	F:GGCGGTAAGGACAACGGGGTGTTTGAATTG R:GAACAACCTAAAACCCGCCTCGCCATCG	Detect VNTRs in STEC [39]	[27]
VNTR-34	(170–172) to (313–314)			F:GACAAGGTTCTGGCGTGTTACCAACGG R:GTTACAACTCACCTGCGAATTTTTTAAGTCCC		
VNTR-9	(474–475) to (613–614)			GCGCTGGTTTAGCCATCGCCTTCTTCC GTGTCAGGTGAGCTACAGCCCGCTTACGCTC		
VNTR-25	(122–124) to (191–192)			GCCGGAGGAGGGTGATGAGCGGTTATATTTAGTG GCGCTG AAAAGACATTCTCTGTTTGGTTTACACGAC		
VNTR-17	(135–136) to (247–248)			GCAGTTGCTCGGTTTTAACATTGCAGTGATGA GGAAATGGTTTACATGAGTTTGACGATGGCGATC		
VNTR-19	(283–284) to (356–357)			GCAGTGATCATTATTAGCACCGCTTTCTGGATGTTC GGGGCAGGGAATAAGGCCACCTGTTAAGC		
VNTR-36	(123–124) to (240–242)			GGCGTCCTTCATCGGCCTGTCCGTTAAAC GCCGCTGAAAGCCCACACCATGC		
VNTR-37	(157–158) to (273–274)			GCCGCCCCTTACATTACGCG GACATTC GCAGGAGAACAACAAAACAGACAGTAATCAGAGCAGC		

The resolution of PCR products of virulence genes, antibiotic resistance genes, and MLVA loci genes were analyzed using the QIAxcel machine at the Department Bacteriology, Mycology, and Immunology, Faculty of Veterinary Medicine, University of Sadat City.

### Statistical analysis

The free online calculator for chi-square at https://www.socscistatistics.com/tests /chisquare2/default2.aspx was utilized to evaluate recovery rates of STEC, the frequencies of serotypes, detection of virulence factors, virulence profiles, efficacies of antimicrobials, multiple antibiotic resistance (MAR) indices, and detection of antimicrobial resistance genes. Phenotypic antimicrobial resistance profiles and associated genes confirmed in this study were changed to binary codes for statistical analysis. Sensitivity to given antimicrobial agent recorded as response 0 and resistance was recorded as response 1. The presence or absence of a specific resistance gene was also scored as 1 or 0, respectively. A heatmap, hierarchical clustering, and Pearson correlation coefficient were calculated using the online tools at https://software.broadinstitute.org/morpheus/. The discriminatory index (DI) was calculated according to the formula of Hunter and Gaston [40]; S = Simpson’s index of diversity, calculated as.

$$ \mathrm{D}=1-1/\mathrm{N}\left(\mathrm{N}-1\right){\sum}_{\mathrm{J}=1}^{\mathrm{S}}\mathrm{Nj}\left(\mathrm{N}\mathrm{j}-1\right) $$

This calculation assesses the probability that MLVA genotyping will assign two randomly tested unrelated serotypes inside the microbial population to different classifications or groups. The online tools at http://insilico.ehu.es/mini_tools /discriminatory_power/index.php were used to calculate the DIs of MLVA loci and their combinations. Moreover, the 95% confidence interval for each DI result was calculated using the free online tools at: http://www.compa ringp artit ions.info/?link=ToolI . A dendrogram based on the MLVA-associated diversity was constructed using the BioNumerics software v. 6.6 (Applied Maths, Sint-Martens-Latem, Belgium).

## Results

### Isolation of STEC and detection of serogroups and serotypes

A total of sixty STEC isolates were identified in the 207 collected samples (29%); these were distributed as follows: 7/15 (50%), 14/20 (70%), 5/10 (50%), 6/12 (50%), 19/50 (38%), and 9/100 (9%) from broilers, duck, cattle meat, cow’s milk, human urine, and human ear discharge, respectively. There was high isolation rates from broilers, duck, cattle meat, cow’s milk, and human urine compared with human ear discharge and a significant difference of *p* < 0.05 was present. A total of twenty-one serogroups and serotypes were identified, the prevalence of serogroup O78 was the highest among the broilers (2 isolates) and ducks (4 isolates) with a rate of 6/60 (10%); O2:H6 was detected in 5/60 (8.3%) of broilers (1 isolate), ducks (2 isolates), and human urine (2 isolates). The O91:H21 was identified in 5/60 (8.3%) of ducks (3 isolates) and cattle (2 isolates); O128:H2 was identified in 3/60 (5%) of ducks (2 isolates) and cattle (1 isolate); and O26:H11 was detected in 3/60 (5%) of ducks (1 isolate) and cattle (2 isolates). In our analysis of human samples, we found that O15:H2 was the highest and identified in 8/60 (13.3%) of human urine (5 isolates) and ear discharge (3 isolates); O17:H18 was detected in 6/60 (10%) of human urine (3 isolates) and ear discharge (3 isolates); O7:H2 in 3/60 (5%) of human urine, and O8:H21 in 5/60 (8.3%) of human urine (2 isolates) and ear discharge (3 isolates). Furthermore, the prevalence of the following strains was the lowest among the obtained strains as follows; O146:H21 in 1/60 (1.6%) of broilers (1 isolate), O1:H7 in 2/60 (3.3%) of broilers (2 isolates), O127:H6 in 2/60 (3.3%) of broilers (1 isolate) and from cattle (1 isolate), O153:H2 in 1/60 (1.6%) from ducks (1 isolate), O121:H7 in 1/60 (1.6%) of ducks (1 isolate), O86 in 1/60 (1.6%) of cattle (1 isolate). Moreover, the following strains; O83, O125:H21, O75, and O124 represented 1/60 (1.6%) of human urine 1 isolate for each. There was a significant difference between the strains exhibiting dissimilar rates with *p* < 0.05 (Table 3).

**Table 3:** Results of the obtained *E. coli* strains from different samples

Strains		Broilers (7)	Duck (14)	Cattle (11)	Human urine (19)	Ear discharge (9)	Total
O146:H21		1					1/60 (1.6%)
O1:H7		2					2/60 (3.3%)
O127:H6		1		1			2/60 (3.3%)
O78		2	4				6/60 (10%)
O2:H6		1	2		2		5/60 (8.3%)
O91:H21			3	2			5/60 (8.3%)
O153:H2			1				1/60 (1.6%)
O128:H2			2	1			3/60 (5%)
O26:H11			1	2			3/60 (5%)
O121:H7			1				1/60 (1.6%)
O86				1			1/60 (1.6%)
O111:H2				2			2/60 (3.3%)
O55:H7				2			2/60 (3.3%)
O15:H2					5	3	8/60 (13.3%)
O17:H18					3	3	6/60 (10%)
O7:H2					3		3/60 (5%)
O8:H21					2	3	5/60 (8.3%)
O83					1		1/60 (1.6%)
O125:H21					1		1/60 (1.6%)
O75					1		1/60 (1.6%)
O124					1		1/60 (1.6%)
Total	21						60/60 (100%)

### Virulence genes and combinations of Shiga toxin genes

**Fig. 1:**
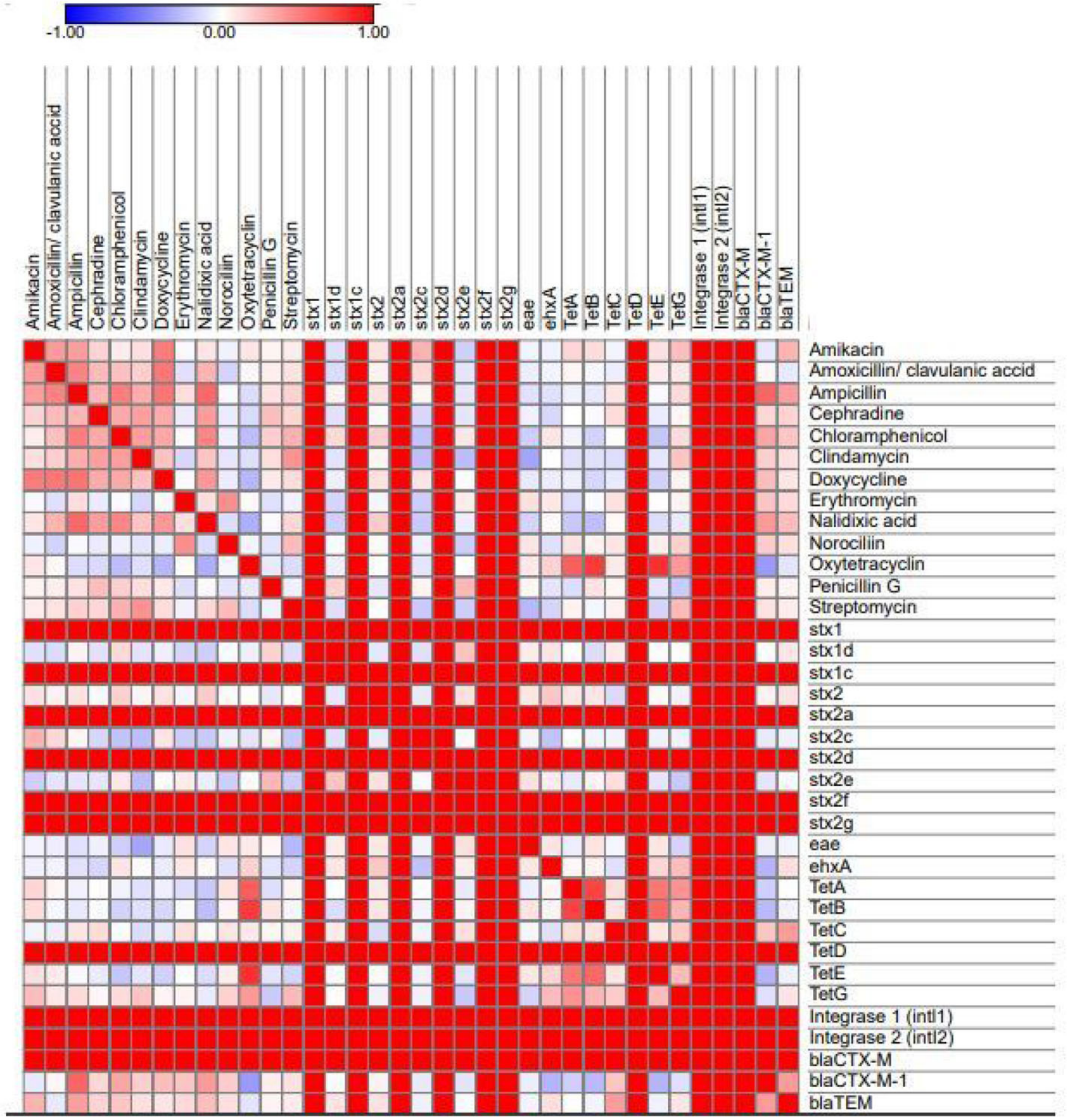
Correlation matrix of phenotypic antimicrobial resistance and antimicrobial resistance genes expressing significant correlations (*p* < 0.05). White squares are not significantly correlated. Red squares indicated significant positive correlation and blue squares show significant negative correlation. The size and strength of color represent the numerical value of the Pearson correlation coefficient

All the STEC strains 60/60 (100%) harbored the *stx*_1_ gene; its subtype *stx*_1d_ was detected in 51/60 (85%), *stx*_2_ gene in 48/60 (80%), *stx*_2c_ subtype in 3/60 (5%), *stx*_2e_ subtype in 42/60 (70%), *eae*A in 2/60 (3.3%), and *ehx*A in 46/60 (76.7%). No *stx*_1c_, *stx*_2a_, *stx*_2d_, *stx*_2f_, or *stx*_2g_ subtypes were detected in any of the isolated STEC strains. As shown in Fig. 1 and Supplementary Table 2, *stx*_1_ was detected in all investigated STEC strains (100%), while the *stx*_1d_ subtype was identified in all broiler and duck STEC isolates (100%). This subtype existed in 72.7, 73.7, and 88.9% of cattle, human urine, and human ear discharge strains, respectively, with highest rate was in human ear discharge strains. Likewise, *stx*_2_ was found in 100, 71.4, 81.8, 78.9, and 77.8% of broiler, duck, cattle, human urine, and human ear discharge strains, respectively, and the highest rate was found in broiler strains. The *stx*_2c_ subtype was identified in 7.1, 9.1, and 5.3% of duck, cattle, and human urine strains, respectively, with the highest estimate was in duck strains. The *stx*_2e_ subtype was identified in broilers and duck STEC strains at 85.7 and 92.9%, respectively. This subtype existed in 54.5, 63.4, and 66.7% of cattle, human urine, and human ear discharge STEC strains, respectively, as the highest rate was found in duck strains. Likewise, *eae*A was detected in 10.5% of the human urine STEC strains while absent elsewhere. The *ehx*A gene was detected in 85.7, 57.1, 63.6, 94.7, and 77.8% of broiler, duck, cattle, human urine, and human ear discharge STEC strains, respectively, with the highest rate was in human urine strains. There found a significant difference among the dissimilar rates of virulence genes *p* < 0.05 (Table 4 and Supplementary Table 1 and Supplementary Fig. 1).

**Table 4:** Results of distribution of virulence genes in STEC isolates from broiler, duck, cattle, and human samples

Origin	Serotype	Virulence genes						
		*stx*_1_	*stx*_1d_	*stx*_2_	*stx*_2c_	*stx*_2e_	*eae*	*ehx*A
Broilers	O146:H21	+*	+	+	-**	+	–	+
Broilers	O1:H7	+	+	+	–	–	–	+
Broilers	O1:H7	+	+	+	–	+	–	+
Broilers	O127:H6	+	+	+	–	+	–	+
Broilers	O78 (2 strains)***	+	+	+	–	+	–	+
Broilers	O2:H6	+	+	+	–	+	–	–
Duck	O91:H21 (2 strains)	+	+	+	–	+	–	+
Duck	O78	+	+	–	–	+	–	+
Duck	O153:H2	+	+	+	+	+	–	–
Duck	O91:H21	+	+	+	–	+	–	–
Duck	O2:H6	+	+	+	–	+	–	–
Duck	O128:H2	+	+	–	–	+	–	–
Duck	O78	+	+	–	–	+	–	–
Duck	O2:H6	+	+	+	–	+	–	+
Duck	O78 (2 strains)	+	+	+	–	+	–	+
Duck	O26:H11	+	+	–	–	–	–	+
Duck	O128:H2	+	+	+	–	+	–	+
Duck	O121:H7	+	+	+	–	+	–	–
Meat	O86	+	+	+	–	+	–	+
Meat	O111:H2	+	+	–	–	+	–	+
Meat	O111:H2	+	+	+	–	–	–	–
Meat	O128:H2	+	+	–	–	–	–	–
Meat	O26:H11	+	–	+	–	+	–	+
Milk	O55:H7	+	+	+	–	+	–	–
Milk	O26:H11	+	+	+	–	–	–	+
Milk	O55:H7	+	+	+	–	–	–	+
Milk	O91:H21	+	–	+	+	–	–	–
Milk	O91:H21	+	–	+	–	+	–	+
Milk	O127:H6	+	+	+	–	–	–	+
Human urine	O15:H2	+	+	+	–	–	–	+
Human urine	O15:H2 (2 strains)	+	+	–	–	–	–	+
Human urine	O15:H2 (2 strains)	+	–	+	–	–	–	+
Human urine	O17:H18	+	+	+	–	+	–	+
Human urine	O17:H18	+	+	–	–	+	–	+
Human urine	O17:H18	+	–	+	–	+	–	+
Human urine	O7:H2 (3 strains)	+	+	+	–	+	–	+
Human urine	O2:H6 (2 strains)	+	+	+	–	+	–	+
Human urine	O8:H21	+	+	+	–	+	–	+
Human urine	O8:H21	+	+	+	–	+	+	+
Human urine	O83	+	–	+	–	–	–	–
Human urine	O125:H21	+	–	+	–	–	–	+
Human urine	O75	+	+	+	–	+	+	+
Human urine	O124	+	+	–	+	+	–	+
Human ear discharge	O8:H21 (3 strains)	+	+	+	–	+	–	+
Human ear discharge	O15:H2 (2 strains)	+	+	+	–	–	–	+
Human ear discharge	O15:H2	+	+	–	–	–	–	–
Human ear discharge	O17:H18 (2 strains)	+	+	+	–	+	–	+
Human ear discharge	O17:H18	+	–	–	–	+	–	–
	Total (percentage)	60/60 (100%)	51/60 (85%)	48/60 (80%)	3/60 (5%)	42/60 (70%)	2/60 (3.3%)	46/60 (76.7%)

(+)* positive, (−)** negative

*** Each STEC strain was obtained from separate sample

We examined the prevalence of combinations of the *stx*_1_, *stx*_2_, and their subtypes; 10 genetic combinations were identified. We found that 31/60 (51.7%) of the strains harbored the *stx*_1_, *stx*_1d_, *stx*_2_, and *stx*_2e_ combination which was the highest among the obtained combinations and 8/60 (13.3%) harbored the *stx*_1_, *stx*_1d_, and *stx*_2_ combination. Both (*stx*_1_ and *stx*_1d_), and (*stx*1, *stx*1d, and *stx*_2e_) combinations were detected in 5/60 (8.3%), for each. Similarly, the combination *stx*1 and *stx*2 was detected in 4/60 (6.7%), while *stx*_1_, *stx*_2_, and *stx*_2e_ combination was detected in 3/60 (5%). The four genetic combinations (*stx*1, *stx*1d, *stx*_2_, *stx*_2c_, and *stx*_2e_), (*stx*_1_, *stx*_2_, and *stx*_2c_), (*stx*_1_ and *stx*_2e_), and (*stx*_1_, *stx*_1d_, *stx*_2c_, and *stx*_2e_) were each present in 1/60 (1.6%) that represented the lowest rate among the gained combinations. The dissimilar rates of combinations were present at significant difference (*p* < 0.05; Table 5). It was clear that many strains harbored many copies of *stx* genes.

**Table 5:** Results of distribution of the *stx*_1_, *stx*_2_, and their subtypes gene combinations in STEC strains

Shiga toxin genes	No. of strains	Percentage
*stx*_1_, *stx*_1d_, *stx*_2_, *stx*_2e_	O146:H21 (1), O1:H7 (1), O127:H6 (1), O78 (4), O91:H21 (3), O2:H6 (5), O128:H2 (1), O86 (1), O17:H18 (3), O7:H2 (3), O8:H21 (5), O121:H7 (1), O55:H7 (1), O75 (1)	31/60 (51.7%)
*stx*_1_, *stx*_1d_, *stx*_2_	O1:H7 (1), O26:H11 (1), O55:H7 (1), O127:H6 (1), O15:H2 (3), O111:H2 (1)	8/60 (13.3%)
*stx*_1_, *stx*_1d_, *stx*_2e_	O78 (2), O111:H2 (1), O17:H18 (1), O128:H2 (1)	5/60 (8.3%)
*stx*_1_, *stx*_1d_, *stx*_2_, *stx*_2c_, *stx*_2e_	O153:H2 (1)	1/60 (1.7%)
*stx*_1_, *stx*_1d_	O26:H11 (1), O15:H2 (3), O128:H2 (1)	5/60 (8.3%)
*stx*_1_, *stx*_2_, *stx*_2c_	O91:H21(1)	1/60 (1.7%)
*stx*_1_, *stx*_2_, *stx*_2e_	O17:H18 (1), O91:H21(1), O26:H11(1)	3/60 (5%)
*stx*_1_, *stx*_2_	O83 (1), O125:H21(1), O15:H2 (2)	4/60 (6.7%)
*stx*_1_, *stx*_2e_	O17:H18 (1)	1/60 (1.7%)
*stx*_1_, *stx*_1d_, *stx*_2c_, *stx*_2e_	O124 (1)	1/60 (1.7%)

### Antimicrobial susceptibility testing

Overall resistance to amikacin, amoxicillin/clavulanic acid, doxycycline, ampicillin, nalidixic acid, and chloramphenicol was detected at 5, 11.7, 16.7, 26.7, 41.7, and 50%, respectively. In this first set of tested antimicrobials the resistance to nalidixic acid and chloramphenicol was more prevalent in the isolates, compared to other antibiotics. While resistance to cephradine, erythromycin, norocillin, oxytetracycline, clindamycin, streptomycin, and penicillin G represented 65, 73.3, 75, 80, 81.7, 91.7, and 96.7%, respectively, for this group of antimicrobials there noticed increased resistance compared with the aforementioned types. We detected significant differences among the efficacies of these antimicrobials with *p* < 0.05 (Supplementary Table 2 and Supplementary Fig. 2).

For broiler and duck strains, the MAR index ranges were 0.38–0.77 and 0.23–0.92, respectively. Furthermore, the MAR index for cattle strains range was 0.38–0.92 and from human urine and ear discharge, the ranges were 0.15–0.85 and 0.31–0.92, respectively. The strains O78 (sample #17), O91:H21 (sample #30), and O17:H18 (sample #58) from duck, cattle, and human ear discharge exhibited the highest MAR indices at 0.92 for each, with a significant difference *p* < 0.05 was present among the dissimilar indices of different strains (Supplementary Table 3).

### Antimicrobial resistance genes

The class 1 and 2 integrons were not detected in any isolates (0.0%), for each, extended-spectrum β-lactamases type *bla*CTX-M and *bla*CTX-M-1 represented 0/60 (0.0%) and 8/60 (13.3%), respectively, and the ampicillin-resistance gene *bla*TEM was identified in 3/60 (5%). The *bla*CTX-M-1 resistance gene expressed the highest distribution pattern among the screened extended-spectrum β-lactamase genes. It was found in 2/7 (28.6%), 2/14 (14.3%), 3/5 (60%), and 1/6 (16.7%) of broiler, duck, cattle meat, and cattle milk STEC strains, respectively, with the highest distribution pattern among cattle meat strains. Moreover, ampicillin-resistance gene *bla*TEM was detected in 2/7 (28.6%) and 1/14 (7.14%) of broiler and duck STEC strains, respectively. For oxytetracycline, the *tet*A, B, E, and G were highly detected among the STEC strains, while *tetC* was the lowest one. The *tet*A was identified in 40/60 (66.7%) of strains it was distributed as follows; 3/7 (42.9%) of broilers, 11/14 (78.6%) of ducks, 3/5 (60%) of cattle meat, 5/6 (83.3%) of cattle milk, 11/19 (57.9%) of human urine, and 7/9 (77.8%) of human ear discharge. The *tet*B gene was identified in 44/60 (73.3%) of strains and distributed as follows; 3/7 (42.9%) of broilers, 11/14 (78.6%) of ducks, 3/5 (60%) of cattle meat, 4/6 (66.7%) of cattle milk, 15/19 (78.9%) of human urine, and 8/9 (88.9%) of human ear discharge. The *tet*C was found in 2/60 (3.3%) of strains and distributed as follows; 1/7 (14.3%) of broilers and 1/14 (7.14%) of ducks. The *tet*E resistance gene was detected in 46/60 (76.7%) of strains and distributed as follows; 1/7 (14.3%) of broilers, 10/14 (71.4%) of ducks, 3/5 (60%), of cattle meat, 5/6 (83.3%) of cattle milk, 19/19 (100%) of human urine, and 8/9 (88.9%) of human ear discharge. The *tet*G was found in 27/60 (45%) of strains and distributed as follows; 3/7 (42.9%) of broilers, 5/14 (35.7%) of ducks, 3/5 (60%) of cattle meat, 4/6 (66.7%) of cattle milk, 7/19 (36.8%) of human urine, and 5/9 (55.6%) of human ear discharge. Finally, the *tet*D was not detected among all the strains. Based on the existence of *tet*A, B, E, and G genes, there found that 10/60 (16.7%) of strains contained no genes, 3/60 (5%) contained one gene, 7/60 (11.7%) harbored two genes, 20/60 (33.3%) expressed three genes, 18/60 (30%) harbored four genes, and 2/60 (3.3%) contained five genes. There was a significant difference among the rates of screened antimicrobial resistance genes with *p* < 0.05 (Supplementary Results Tables 4 and 5).

### Associations between isolation source, strain, and phenotypic and genotypic characters

**Fig. 2:**
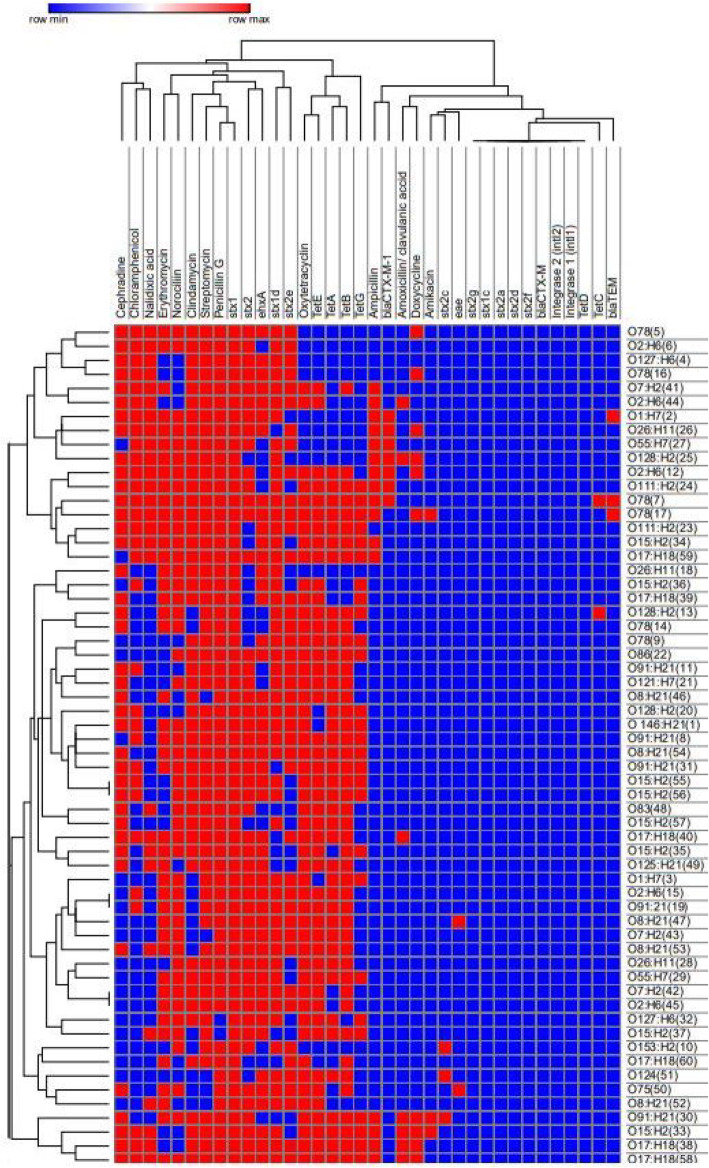
Heatmap and hierarchical clustering of *E. coli* isolates to 5 clusters based on their phenotypic (antimicrobial resistance), genotypic (antimicrobial resistance genes), and virulence genes expressing differences between isolates. Red represented presence and blue represented absence of phenotypic resistance, resistance genes, and virulence genes. Hierarchical clustering was performed using Wald’s method and a binary distance matrix

The relationship of the existence of antimicrobial resistance, resistance genes, strain, and the source of samples was discovered to detect possible associations among the isolates. Phenotypic antimicrobial resistance profiles and associated genes confirmed in this study were changed to binary codes for statistical analysis. Sensitivity to given antimicrobial agent recorded as response 0 and resistance was recorded as response 1. The presence or absence of a specific resistance gene was also scored as 1 or 0, respectively. The Pearson correlation coefficient was calculated using the online tools at https://software.broadinstitute.org/morpheus/. Some strains from broilers and ducks exhibited high virulence, phenotypic and genotypic antimicrobial resistance (Fig. 1). There was a significant difference (*p* < 0.05) in strains impact on virulence genes, phenotypic antimicrobial resistance, and resistance genes (Fig. 2). Correlation matrix analysis (Fig. 1) and hierarchical clustering with heat map (Fig. 2) were utilized to detect associations between the phenotypic and genotypic features and origin of the strains. Correlation analysis showed positive relationships among the presence of resistance to β-lactams especially to ampicillin and the presence of β-lactamase genes as the *bla*CTX-M-1 and *bla*TEM (Fig. 2, *p* < 0.05). Significant positive correlations of antibiotic resistances proved co-occurrence of resistance may be predominant, (*p* < 0.05) and confirmed the presence of multiple-drug-resistant strains (MDR). For example, resistance to amoxicillin/clavulanic acid and ampicillin were positively correlated with resistance to amikacin, cephradine, clindamycin, doxycycline, and nalidixic acid resistances tested. The existence of the *bla*CTX-M-1 and *bla*TEM were positively correlated with resistance to chloramphenicol and nalidixic acid (*p* < 0.05).

### Analysis of the MLVA loci

The data presented in Supplementary Results Table 6 revealed that all strains identified by the three loci VNTR 3, 25, and 36 included three different alleles. Furthermore, the remaining five loci (VNTR 9, 17, 19, 34, and 37) divided all the STEC strains into two different alleles. Diversity analysis revealed that the discriminatory power of the used loci differed greatly, with discriminatory indices (DIs) ranged from 0.216 to 0.613. The allelic profile of each strain with every MLVA locus was recorded and compared among all the 60 STEC strains. Based on these results, every strain has its own specific allelic profile or code which was named a genotype. The strains expressing the same allelic profiles were included in the same genotype. And after this allelic profile differentiation a total of 26 different genotypes (GTs) were found using all the 8 MLVA loci with DI = 0.9277. The locus VNTR 3 provided the highest discriminatory power (DI > 0.6) while the loci VNTR 9, 17, 19, 36 and 37 proved moderate discriminatory power (0.3<DI<0.6); the loci VNTR 25 and VNTR 34 had only limited discriminatory power (DI < 0.3). The selected loci were highly predictive for discrimination among strains with a 95% confidence interval (CI) 1.000–1.000.

### The prevalence of specific MLVA profiles and genotypes (GT) among strains

A total of 26 genotypes were obtained, the genotype GT 21 was the most prevalent; it was identified in 12/60 (20%) of strains. GT 22 was identified in 6/60 (10%), GT 2 in 5/60 (8.3%), and GT 6 in 5/60 (8.3%). The genotypes GT 3 and GT 19 were detected in 3/60 (5%) and 4/60 (6.7%) of strains, respectively, while the GT10, 11, 20, and 24 were each identified in 2/60 (3.3%). The genotypes GT 1, 5, 7, 8, 9, 12, 13, 14, 15, 16, 17, 18, 23, 25, and 26 were each identified in 1/60 (1.7%) which represented the lowest prevalence rate. There was a significant difference between the genotypes with different rates *p* < 0.05 (Supplementary Table 7 and Supplementary Fig. 3).

### Evaluation of the discriminatory power of MLVA loci combinations

**Fig. 3:**
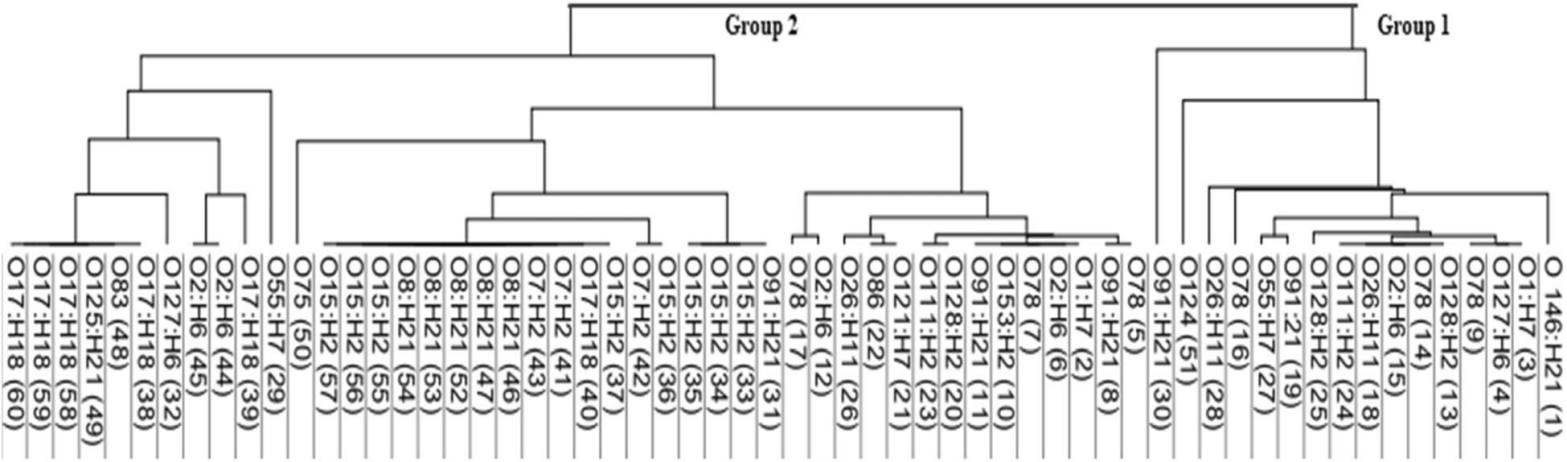
Dendrogram of STEC strains based on their multiple-locus variable-number tandem-repeat analysis (MLVA) profiles. Strains are clustered into two groups, group 1 less complex and included ten types; and group 2 more complex and contained sixteen clustered genotypes

The 60 STEC strains were resolved to 26 different genotypes with a DI of 0.9277 using 8 MLVA loci, which were clustered into 24 diverse clustered types, five unique types, and 19 clustered types. The clusters contained 1 (*n* = 1) to 2 (*n* = 12) analogous strains (Supplementary Table 8), and the 23 diverse types were classified into two groups (Fig. 3). Group 1 was less complex and included nine clustered types; by contrast group 2 was more complex and contained fifteen clustered types. The discrimination power was compared among different MLVA combinations. The first combination included VNTR 3, 9, 17, 19, 36, and 37 detected 26 genotypes in 18 clusters with a DI of 0.9138. The second combination included VNTR 25 and 34, which resolved the 60 strains into 26 genotypes which were categorized into five clusters with a DI of 0.6161. Hence, the six loci VNTRs 3, 9, 17, 19, 36, and 37 alone are sufficiently robust for preliminary molecular epidemiological studies when rapid results are of paramount importance. The implemented MLVA combinations were capable of discriminating among STEC strains with a 95% confidence interval (CI) of 1.000–1.000.

## Discussion

The STEC strains such as O157 and non-O157 have acquired genetic traits that made them potential risk for human. In humans, STEC causes a wide range of infections including diarrhea, complicated hemorrhagic colitis (HC), HUS, and thrombotic thrombocytopenic purpura. Humans may acquire infection as a result of contamination of food and water [41, 42]. Our first goal was to identify STEC from multiple sources. Our most prominent source was duck fecal samples; STEC strains were isolated from 14/20 (70%) samples. These results are nearly similar to published results from Malaysia during 2012 [43], which confirmed the existence of STEC in duck feces with a frequency of 87.93%. Moreover, we isolated STEC from 50% of the broilers; this result was smaller than reported results from Egypt during 2015 [44], which proved that *E. coli* was present in 75% of local broilers. Our isolation rate from cow’s milk was 50% similar to results from Egypt during 2019 [45], which confirmed the existence of *E. coli* in 50% of the composite milk samples from both cows and buffaloes. We isolated STEC from 50% of the cattle meat samples, which was higher than Gwida et al. [46] who identified *E. coli* in 27% of raw beef. These results confirmed that the STEC could be isolated from a wide variety of food animals and poultry found in small farms within rural localities [47]. Likewise, our STEC isolation from human urine samples was lesser than Córdoba et al. [48], who identified *E. coli* from patients of suspected urinary tract infections in primary care, at Denmark 2017. The isolation from human ear discharge was lesser than Kibret and Abera [49]. Among the strains from broiler and duck, O78 was the most prevalent, detected in 6/60 (10%) which was similar to Wang et al. [50] and Abd El Tawab et al. [51] who confirmed the high prevalence of O78 among strains that cause avian colibacillosis. Both O2 and O128 strains were previously reported in poultry by Byomi et al. [52] and in ducks as reported by El-Shabrawy et al. [53], moreover the existence of O121:H7 and O146 strains in chicken was confirmed by Enany et al. [54], while recorded in ducks by Wang et al. [50]. The distribution of O1, O26:H11, O91, O127, and O153 strains in samples from chickens and ducks were similar to Abd El-Mongy et al. [44] and Wang et al. [50]. The O26:H11, O91:H21, O121:H7, O128:H2, O153:H2, O1:H7, O2:H6, O78, O127:H6, and O146:H21 [50–55] are serious avian STEC strains that cause severe localized or extraintestinal infections. The extraintestinal infection called colibacillosis which affects broiler chickens from 4 to 6 weeks of age and distinguished by causing acute fatal septicemia or sub-acute airsacculitis, fibrinous pericarditis, peritonitis, and salpingitis [54]. The O26:H11, O111:H2, and O128:H2 strains have all been identified previously in cattle meat products [56]. These strains from meat are implicated in the etiopathogenesis of calve diarrhea either mild or sever with significant alterations in the animal hematological and biochemical parameters, moreover these strains exhibited public health importance [57]. Furthermore, the isolation of O55:H7, O86, O91:H21, and O127:H6 from cases of bovine mastitis worldwide was confirmed [58]. Interestingly, the isolation of O2:H6, O15:H2, O7:H2, O17:H18, O8:H21, O83, O125:H21, O75, and O124 in association with human urinary tract infections suggests that these strains may be uropathogenic [59]. We found no published reports documenting the serotyping of STEC associated with otitis media; here, we found that O8:H21, O15:H2, and O17:H18 were the most prevalent serotypes. The non-O157 STEC are more frequently isolated with about 4-fold higher isolation rate than O157, from HUS cases the non-O157 STEC proportion varying from 7 to 90% [60]. The enteropathogenic *E. coli* serogroups are human-specific pathogens which cause endothelial infection, intestinal thrombotic microangiopathy, and ischemic enteritis [61]. The O1:H7, O2:H6, O7:H2, O8:H21, O15:H2, O17:H18, O26:H11, O75, O83, O91:H21, O121:H7, O124, O125, O153:H2, and O128:H2 strains imparted zoonotic impact and have been isolated from human infections [62]. The O55, O86, O111, and O127 strains have been considered as major causes of acute and persistent infantile diarrhea in many developing countries [62, 63]. The O1:H7, O2:H6, O8:H21, O15:H2, O55:H7, O75, O91:H21, O111:H2, and O128:H2 STEC strains have been isolated from HUS patients [62]. The Shiga toxin gene, *stx*_1_, was detected in all strains, while *stx*_2_ was detected in 80% of strains; the increased detection rate from broilers, ducks, beef, and human urine samples agree with the recent report from Egypt during the year 2020 [64]. Moreover, the high existence patterns of both *stx*_1_ and *stx*_2_ from cattle milk strains were confirmed by Ranjbar et al. [65]. Of the *stx*_1_ subtypes, *stx*_1d_ was detected most prominently in 85% of the isolates, while *stx*_1c_ subtype was not detected at all. Of all the *stx*_1_ positive strains, *stx*_1d_ subtype was detected predominantly in our isolates from chicken and duck (100%) and was identified in 73.7 and 72.7% of the isolates from humans and cattle, respectively. From these results, the *stx*_1d_ was commonly found in animal strains as confirmed by Kumar et al. [66], while its high distribution among human strains wasn’t confirmed by him, but he stated that it could be linked with a mild course of disease. Moreover, the high distribution of the *stx*_1d_ subtype in human strains come in contradiction with that reported by EFSA BIOHAZ Panel et al. [67], those confirmed that the *stx*_1d_ subtype wasn’t predominantly associated with hospitalisations and bloody diarrhea. As the human cases were cattle farm workers and farmers rearing broilers and ducks on the small scale, this result could be regarded to the mobile genetic elements like bacteriophages, insertion sequence elements, pathogenicity islands, plasmids, and transposons that play a vital role in the evolution of human STEC and changing them to *stx*_1d_ subtype positive [67]. The *stx*_2_ subtypes were screened and *stx*_2c_ subtype prevalence was 7.1, 9.1, and 5.3% in duck, cattle, and human urine STEC, respectively. The prevalence of this subtype from duck strains contradicts with the result from India during the year 2009 [68], which proved the absence of *stx*_2c_ subtype in duck STEC strains. Moreover, the high prevalence of this subtype in cattle STEC than human STEC agrees with the report from Brazil during the year 2006 [69]. The *stx*_2e_ subtype existed in broilers and duck STEC strains at 85.7 and 92.9%, respectively. This subtype existed in 54.5, 63.4, and 66.7% of cattle, human urine, and human ear discharge STEC strains, respectively. The prevalence of this subtype in broilers and ducks contradicts with published data from china during 2012 [70], which confirmed that chicken *E. coli* isolates harbored no *stx*_2e_ subtype. Furthermore, the distribution of this subtype in cattle meat and milk STEC exceeded that recorded in a report from Germany during the year 2011 [71], while its existence in human cases was confirmed by EFSA BIOHAZ Panel et al. [67]. The *eae*A was detected in 3.3% of isolates, which was smaller than Elsayed and Munir [64]. Although we used a universal oligonucleotide primer pair EAE-F and EAE-RB with homology to the 3’variable region of *eae* (that detects all types of eae variants described at the moment of the manuscript published by Blanco et al. [36], who screened 514 STEC isolates for *stx* and *eae* genes and their subtypes, there found that most of our STEC isolates harbored no *eae* gene which shows similarity to his results. But two O26:H- of his STEC isolates harbored *eae* type β1, while one isolate O26:H11 contained no *eae* that confirms the possibility of existence of O26:H11 negative for *eae*, added to that, he confirmed three isolates of O111:H- contained γ2 type of *eae*, which is quite different from from our O111:H2. It is clear that most STEC infections with serious complications like hemolytic uremic syndrome are caused by bacteria that attach to and efface enterocytes and harbor active *eae* gene. However, HUS has been connected to a subset of STEC isolates that do not possess *eae* genes [18]. The *ehx*A was detected in 77% of isolates, a rate surpassed that of Elsayed and Munir [64]. The existence of Shiga toxin genes and virulence genes in most of isolated STEC strains from animals and human represent a serious problem to public health as these strains could result in dangerous infections and could transmit these characters to other pathogenic and nonpathogenic bacterial agents through the mobile genetic elements [17, 67]. Of note, to the best of our knowledge, this is the first report of the specific prevalence of given combinations of the *stx* genes; then 10 combinations featured here are novel and not previously reported [14, 66], these combinations were recorded to express the more frequently found *stx* genes among the gained STEC strains which can differentiate between them. From the distribution patterns of *stx* gene combinations, it was clear that several strains harbored many copies of *stx* genes, as some combinations composed of 4 copies as *stx*_1_, *stx*_1d_, *stx*_2_, *stx*_2e_ which represented 51.7% of the strains and 5 as *stx*_1_, *stx*_1d_, *stx*_2_, *stx*_2c_, *stx*_2e_ which represented 1.7% of the obtained strains. Although our *stx* gene copies were high, this fact comes in agreement with the published data from Finland during 2002 [15], which confirmed the existence of 11 *stx* gene combinations; the most prevalent combinations were *stx*_2_ with *stx*_2c_ estimated (42%) which contained two copies. And from the O157 strains, 64% carried *stx*_2_ with *stx*_2c_ versus 2% of the STEC strains. Furthermore, the existence of multiple copies of *stx* genes in human strains was confirmed by a report from Germany during 2006 [72], which proved that the genotype *stx*_2d_-_activatable_, *stx*_1_, and *stx*_2_ that contains 3 copies of *stx* genes was present in 8/60 (13.3%) of STEC isolates.

The STEC strains were screened against a panel of 13 antimicrobial agents that were selected on the basis of their medical importance. Prioritization criterion 1 (P1): an antimicrobial used widely among patients with critical infections and in bacterial diseases in health care settings for which this antimicrobial class is the only or one of few alternatives available. Prioritization criterion 2 (P2): an antimicrobial used widely and of the class that may be useful for treating critical infections in health care settings but whose use may favor the generation of resistance. Prioritization criterion 3 (P3): The antimicrobial class typically chosen to control infections in those infected with resistant bacteria or bacteria that harbor resistance genes from non-human origins [73].

Our isolates showed varying susceptibilities to antimicrobial agents. Among our findings, 73% of the isolates were resistant to erythromycin which was lower than Rubab and Oh [74]; by contrast, the rates of resistance to nalidixic acid, oxytetracycline, and streptomycin were higher than him. The extent of resistance to ampicillin was lesser than Rubab and Oh [74], and the rate of resistance to chloramphenicol outpaced that of Elsayed and Munir [64]. The resistance observed to cephradine, norocillin, and penicillin G exceeded that of Elsayed and Munir [64], and the resistance to doxycycline was lower than him. The extent of resistance to amoxicillin/clavulanic acid was smaller than Elsayed et al. [38] and the resistance to clindamycin was greater than Rubab and Oh [74]. Several isolates expressed multidrug-resistant (MDR) and extensively drug-resistant (XDR) phenotypes. The correlation analyses proved co-occurrence of resistance to various antimicrobials, exemplifies a significant concern for animal and human medicine alike. Moreover, resistance to some antimicrobials was linked with susceptibility to others. As clear, erythromycin resistance was related with susceptibility to amikacin, amoxicillin/clavulanic acid, chloramphenicol, doxycycline, and oxytetracycline. This finding is remarkable because when discussing MDR or XDR STEC it may promote the selection of alternative antimicrobials. Most of the strains exhibited MAR indices that surpassed 0.2; these results suggest that these serotypes may have originated from high-risk sources with uncontrolled implementation of antimicrobial agents [75]. Inadquate antimicrobial selection and abuse can lead to resistance in different bacteria and make it more difficult to treat bacterial infections [76]. High frequencies of antimicrobial drug resistance were observed in STEC strains recovered from the collected samples, which was confirmed to be more common in non-O157 isolates and could contribute to serious disease outcomes [77]. Many antimicrobials were implemented for food producing animals as growth promoters and for prevention, control, and treatment of diseases. From these types, tetracyclines, penicillin, and cephalosporins were used, that represent public health hazards. This uncontrolled use of antimicrobials results in emergence of antimicrobial resistance, hypersensitivity, carcinogenicity, bone marrow depression, mutagenicity, teratogenicity, and disturbance of intestinal normal flora [78, 79]. The extended-spectrum β-lactamases (ESBLs) genes mediate the production of enzymes that destroy antimicrobials belonging to the penicillin and cephalosporin classes and turn them ineffective [80]. The bacterial pathogens can carry antimicrobial resistance genes on mobile elements. These elements can be horizontally transferred to various bacterial species, which could change the recipient strain to become drug resistant. The *Kluyvera* spp. are environmental saprophytes that considered the source of *bla*_CTX-M_. The *CTX*-M-type genes are the most common ESBL genes found in *E. coli* and *Klebsiella pneumoniae* isolates responsible for the worldwide community- and hospital-acquired infections. Moreover, the TEM- genes are also found in various types of environmental saprophytes [21].

The *bla*CTX-M-1 was detected in 13.3% of isolates, which was confirmed in 2/7 (28.6%) of broilers and was lower than the published results by Kim et al. [81]. It was detected in 2/14 (14.3%) of duck strains which was lesser than recent records from India during 2020 [82], while its existence in cattle meat comes in contradiction with Kennedy et al. [83], who proved its absence in STEC strains from abattoir. Additionally, its presence in the STEC from raw milk was confirmed by Ahmed et al. [84]. The ampicillin-resistance gene *bla*TEM was found in 2/7 (28.6%) of the *stx* positive isolates of broilers that contradicts with Saad et al. [85], who confirmed its existence in *stx* negative *E. coli* from chicken, while the existence of *bla*TEM in STEC strains from duck was confirmed by Kim et al. [81].

The selected *tet*A, B, C, D, E, and G oxytetracycline resistance genes encode for efflux proteins present in the cytoplasmic membrane of gram-negative bacteria. They work as antiporters exchanging a proton for a monocationic magnesium-tetracycline complex and reducing the tetracycline amount in the bacterial cytoplasm [86, 87]. In Egypt, there found low number of reports that examined the existence of tetracycline resistance genes in *E. coli* isolated from a large variety of animals and human samples with various histories of exposure to tetracyclines. In our study, the *tet*A and B exhibited high distribution patterns among animal and human strains as confirmed by Bryan et al. [88]. The existence of *tet* C in chicken comes similar to Bryan et al. [88], while its existence in duck isolates differs. Although we gained high distribution patterns of *tet* E and G among animal and human strains, *tet*D was absent, these results come in contradiction with Bryan et al. [88], who found no *tet* E and G among animal and human isolates and confirmed existence of *tet*D gene among human isolates. Although Bryan et al. [88] found that 22.2 and 1.9% of the isolates contained two and three *tet* genes, respectively, our results surpassed him in the number of contained genes as we found that 7/60 (11.7%) harbored two genes, 20/60 (33.3%) expressed three genes, 18/60 (30%) harbored four genes, and 2/60 (3.3%) contained five genes. And the rational interpretation of this fact could be regarded to the strong selection pressures of environments contained high levels of tetracycline that results in acquisition of several tetracycline resistance genes [88]. In this study the identification of resistance genes emphasizes the fact that STEC can serve as reservoirs for antimicrobial resistance genes that could be passed to pathogenic microorganisms that infect humans.

This study is one of the first to consider the applicability of using the eight MLVA loci described by Izumiya et al. [28] for molecular characterization and genotyping of STEC strains in Egypt. The data presented add to our understanding of the genetic diversity and relatedness of various STEC strains. The methodology will facilitate comparisons among distinct genetic profiles with respect to the origin of host and geographic locations. The MLVA analysis technique was chosen based on technical and financial considerations as well as the high discriminatory power [23, 26]. The VNTR 3, 9, 17, 19, and 36 loci were the most polymorphic and permitted us to evaluate our strains with increased resolution. VNTR 3, 9, 17, 25, and 36 exhibited high discriminatory indices and allele numbers when compared with the same loci evaluated in *E. coli* strains O26, O111, and O157 by Izumiya et al. [28]. The DI of VNTR 34 was similar to that of Izumiya et al. [28] although the allele number was lower. Here, VNTR 19 exhibited a greater DI and a lesser allele number, VNTR 37 exhibited a smaller DI and an increased allele number compared with the same loci evaluated by Izumiya et al. [28]. The MLVA typing using the eight aforementioned loci VNTR 3, 9, 25, 17, 19, 34, 36, and 37 provided discriminatory genotyping for the 60 strains. Our results revealed 26 distinct genotypes with different allelic profiles; the efficacy of this methodology surpassed that based on *stx* gene combinations. As such, our results concur with those of previous studies as we found that MLVA is capable of high discriminatory power that surpasses the serotype grouping via PCR amplification of virulence genes [89].

## Conclusions

In conclusion, the STEC strains are widely distributed in broilers, duck, cattle, and human infections. This is the first report of a detailed detection of virulence repertoire, phenotypic and genotypic antimicrobial resistance, and MLVA typing of STEC strains from different sources in Egypt. The gained isolates showed lowered resistance to amikacin, amoxicillin/clavulanic acid, doxycycline, ampicillin, nalidixic acid, and chloramphenicol. Furthermore, the uncontrolled use of antibiotics for STEC infections in animals represents a potential risk for public health. There was a significant impact of serogroups and serotypes on virulence genes, antimicrobial resistance, and resistance genes. MLVA typing considered useful genotyping method and the results from the VNTR 3, 9, 17, 19, 36, and 37 loci are sufficiently robust that they can be used for preliminary molecular epidemiological studies. Most of the animal STEC strains were not found in human infections except the O2:H6 which expressed different MLVA profiles. The obtained results will be useful toward controlling STEC; with these methods it will be comparatively easy to assess linked clusters, cluster growth, and transmission dynamics.

## Supplementary Information

**Additional file 1 S1.** Results of distribution patterns of virulence genes among various *E. coli* strains. **S2.** Antimicrobial susceptibility patterns of 60 *E. coli* strains from different sources. **S3.** Results of multiple antimicrobial resistance indices of various strains from different sources. **S4.** Results of distribution of class 1 and 2 integrons, extended-spectrum β-lactamase, and ampicillin-resistance genes in various strains. **S5.** Results of the distribution patterns of oxytetracycline resistance genes among the phenotypically resistant strains. **S6.** Results of the gained tandem repeats with the utilized MLVA loci and the discriminatory index. **S7.** Results of genotypes and allelic profiles of various STEC strains after MLVA typing. **S8.** Evaluation of the discriminatory power of different MLVA loci combinations.

## Data Availability

The data and material are available in the manuscript and the supplementary material.

## References

[1] Valilis E, Ramsey A, Sidiq S, DuPont HL (2018). Non-O157 Shiga toxin-producing *Escherichia coli*-a poorly appreciated enteric pathogen: systematic review. Int J Infect Dis.

[2] Gyles CL (2007). Shiga toxin-producing *Escherichia coli*: an overview. J Anim Sci.

[3] Gonzalez AGM, Cerqueira AMF (2020). Shiga toxin-producing Escherichia coli in the animal reservoir and food in Brazil. J Appl Microbiol.

[4] Cavalcanti AMF, Hernandes RT, Takagi EH, Guth BEC, Ori ÉL, Pinheiro SRS, Andrade TS, Oliveira SL, Cergole-Novella MC, Francisco GR, Santos LFD (2020). Virulence profiling and molecular typing of Shiga toxin-producing *E. coli* (STEC) from human sources in Brazil. Microorganisms.

[5] Zhang G, Meredith TC, Kahne D (2013). On the essentiality of lipopolysaccharide to gram-negative bacteria. Curr Opin Microbiol.

[6] Aguiniga LM, Yaggie RE, Schaeffer AJ, Klumpp DJ (2016). Lipopolysaccharide domains modulate urovirulence. Infect Immun.

[7] Paton JC, Paton AW (1998). Pathogenesis and diagnosis of Shiga toxin-producing *Escherichia coli* infections. Clin Microbiol Rev.

[8] Lacher DW, Gangiredla J, Patel I, Elkins CA, Feng PC (2016). Use of the *Escherichia coli* identification microarray for characterizing the health risks of Shiga toxin-producing *Escherichia coli* isolated from foods. J Food Prot.

[9] Bai X, Fu S, Zhang J, Fan R, Xu Y, Sun H, He X, Xu J, Xiong Y (2018). Identification and pathogenomic analysis of an *Escherichia coli* strain producing a novel Shiga toxin 2 subtype. Sci Rep.

[10] Hughes AC, Zhang Y, Bai X, Xiong Y, Wang Y, Yang X, Xu Q, He X (2019). Structural and functional characterization of Stx2k, a new subtype of Shiga toxin 2. Microorganisms..

[11] DeGrandis S, Law H, Brunton J, Gyles C, Lingwood CA (1989). Globotetraosylceramide is recognized by the pig edema disease toxin. J Biol Chem.

[12] Schmitt CK, McKee ML, O'Brien AD (1991). Two copies of Shiga-like toxin II-related genes common in enterohemorrhagic Escherichia coli strains are responsible for the antigenic heterogeneity of the O157:H- strain E32511. Infect Immun.

[13] Law D (2000). Virulence factors of Escherichia coli O157 and other Shiga toxin-producing E. coli. J Appl Microbiol.

[14] Dong HJ, Lee S, Kim W, An JU, Kim J, Kim D, Cho S (2017). Prevalence, virulence potential, and pulsed-field gel electrophoresis profiling of Shiga toxin-producing *Escherichia coli* strains from cattle. Gut Pathog.

[15] Eklund M, Leino K, Siitonen A (2002). Clinical *Escherichia coli* strains carrying stx genes: stx variants and stx-positive virulence profiles. J Clin Microbiol.

[16] Imamovic L, Muniesa M (2011). Quantification and evaluation of infectivity of Shiga toxin-encoding bacteriophages in beef and salad. Appl Environ Microbiol.

[17] Martínez-Castillo A, Muniesa M (2014). Implications of free Shiga toxin-converting bacteriophages occurring outside bacteria for the evolution and the detection of Shiga toxin-producing Escherichia coli. Front Cell Infect Microbiol.

[18] Newton HJ, Sloan J, Bulach DM, Seemann T, Allison CC, Tauschek M, Robins-Browne RM, Paton JC, Whittam TS, Paton AW, Hartland EL (2009). Shiga toxin-producing Escherichia coli strains negative for locus of enterocyte effacement. Emerg Infect Dis.

[19] Schroeder CM, White DG, Meng J (2004). Retail meat and poultry as a reservoir of antimicrobial-resistant *Escherichia coli*. Food Microbiol.

[20] Karama M, Mainga AO, Cenci-Goga BT, Malahlela M, El-Ashram S, Kalake A (2019). Molecular profiling and antimicrobial resistance of Shiga toxin-producing Escherichia coli O26, O45, O103, O121, O145 and O157 isolates from cattle on cow-calf operations in South Africa. Sci Rep.

[21] Raphael E, Wong LK, Riley LW (2011). Extended-spectrum Beta-lactamase gene sequences in gram-negative saprophytes on retail organic and nonorganic spinach. Appl Environ Microbiol.

[22] Michalova E, Novotna P, Scheleglova J (2004). Tetracyclines in veterinary medicine and bacterial resistance to them. Vet Med Czech.

[23] Keys C, Kemper S, Keim P (2005). Highly diverse variable number tandem repeat loci in the *E. coli* O157:H7 and O55:H7 genomes for high resolution molecular typing. J Appl Microbiol.

[24] Lindstedt BA, Brandal LT, Aas L, Vardund T, Kapperud G (2007). Study of polymorphic variable-number of tandem repeats loci in the ECOR collection and in a set of pathogenic *Escherichia coli* and *Shigella* isolates for use in a genotyping assay. J Microbiol Methods.

[25] Gorgé O, Lopez S, Hilaire V, Lisanti O, Ramisse V, Vergnaud G (2008). Selection and validation of a multilocus variable-number tandem-repeat analysis panel for typing *Shigella* spp. J Clin Microbiol.

[26] Noller AC, McEllistrem MC, Pacheco AG, Boxrud DJ, Harrison LH (2003). Multilocus variable-number tandem repeat analysis distinguishes outbreak and sporadic Escherichia coli O157:H7 isolates. J Clin Microbiol.

[27] Jenke C, Harmsen D, Weniger T, Rothganger J, Hyytia-Trees E, Bielaszewska M, Karch H, Mellmann A (2010). Phylogenetic analysis of enterohemorrhagic *Escherichia coli* O157, Germany, 1987-2008. Emerg Infect Dis.

[28] Izumiya H, Pei Y, Terajima J, Ohnishi M, Hayashi T, Iyoda S, Watanabe H (2010). New system for multilocus variable-number tandem-repeat analysis of the enterohemorrhagic Escherichia coli strains belonging to three major serogroups: O157, O26, and O111. Microbiol Immunol.

[29] Krüger A, Lucchesi PM, Sanso AM, Etcheverría AI, Bustamante AV, Burgán J, Fernández L, Fernández D, Leotta G, Friedrich AW, Padola NL, Rossen JW (2015). Genetic characterization of Shiga toxin-producing Escherichia coli O26:H11 strains isolated from animal, food, and clinical samples. Front Cell Infect Microbiol.

[30] Feng P, Weagant S D, Grant M A, Burkhardt W. Bacteriological analytical manual chapter 4 enumeration of *Escherichia coli* and the coliform bacteria, U.S. Food and Drug Administration 10903 New Hampshire Avenue Silver Spring, MD 209931–888-INFO-FDA (1–888–463-6332) (2015).

[31] Quinn P, Bryan K, Finola C, Hartigan P, Fitzpartrick ES (2011). Veterinary microbiology and microbial diseases.

[32] Müller EE, Ehlers MM (2005). Biolog identification of non-sorbitol fermenting bacteria isolated on *E. coli* O157 selective CT-SMAC agar. Water SA.

[33] Clinical and Laboratory Standards Institute (2014). Performance standards for antimicrobial susceptibility testing; twenty-fourth informational supplement CLSI document M100-S24.

[34] Zhang W, Bielaszewska M, Kuczius T, Karch H (2002). Identification, characterization, and distribution of a Shiga toxin 1 gene variant (stx (1c)) in *Escherichia coli* strains isolated from humans. J Clin Microbiol.

[35] Scheutz F, Teel LD, Beutin L, Piérard D, Buvens G, Karch H, Mellmann A, Caprioli A, Tozzoli R, Morabito S, Strockbine NA, Melton-Celsa AR, Sanchez M, Persson S, O'Brien AD (2012). Multicenter evaluation of a sequence-based protocol for subtyping Shiga toxins and standardizing Stx nomenclature. J Clin Microbiol.

[36] Blanco M, Blanco JE, Mora A, Dahbi G, Alonso MP, González EA, Bernárdez MI, Blanco J (2004). Serotypes, virulence genes, and intimin types of Shiga toxin (verotoxin)-producing *Escherichia coli* isolates from cattle in Spain and identification of a new intimin variant gene (eae-xi). J Clin Microbiol.

[37] Ng LK, Martin I, Alfa M, Mulvey M (2001). Multiplex PCR for the detection of tetracycline resistant genes. Mol Cell Probes.

[38] Elsayed MSAE, Roshdey T, Salah A, Tarabees R, Younis G, Eldeep D (2019). Phenotypic and genotypic methods for identification of slime layer production, efflux pump activity, and antimicrobial resistance genes as potential causes of the antimicrobial resistance of some mastitis pathogens from farms in Menoufia, Egypt. Mol Biol Rep.

[39] Hyytiä-Trees E, Smole SC, Fields PA, Swaminathan B, Ribot EM (2006). Second generation subtyping: a proposed PulseNet protocol for multiple-locus variable-number tandem repeat analysis of Shiga toxin-producing *Escherichia coli* O157 (STEC O157). Foodborne Pathog Dis.

[40] Hunter PR, Gaston MA (1988). Numerical index of the discriminatory ability of typing systems: an application of Simpson's index of diversity. J Clin Microbiol.

[41] Luna-Gierke RE, Griffin PM, Gould LH, Herman K, Bopp CA, Strockbine N, Mody RK (2014). Outbreaks of non-O157 Shiga toxin-producing *Escherichia coli* infection: USA. Epidemiol Infect.

[42] Allende A, Monaghan J (2015). Irrigation water quality for leafy crops: a perspective of risks and potential solutions. Int J Environ Res Public Health.

[43] Adzitey F, Liew CY, Aronal AP, Huda N (2012). Isolation of *Escherichia coli* from ducks and duck related samples. Asian J Anim Vet Adv.

[44] Abd El Tawab AA, Ammar AM, Nasef SA, Reda RM (2015). Prevalence of *E. coli* in diseased chickens with its antibiogram pattern. Banha Vet Med J.

[45] Fahim KM, Ismael E, Khalefa HS, Farag HS, Hamza DA (2019). Isolation and characterization of *E. coli* strains causing intramammary infections from dairy animals and wild birds. Int J Vet Sci Med.

[46] Gwida M, Hotzel H, Geue L, Tomaso H (2014). Occurrence of Enterobacteriaceae in raw meat and in human samples from Egyptian retail sellers. Int Sch Res Notices.

[47] Amézquita-López BA, Quiñones B, Cooley MB, León-Félix J, Castro-del Campo N, Mandrell RE, Jiménez M, Chaidez C (2012). Genotypic analyses of Shiga toxin-producing *Escherichia coli* O157 and non-O157 recovered from feces of domestic animals on rural farms in Mexico. PLoS One.

[48] Córdoba G, Holm A, Hansen F, Hammerum AM, Bjerrum L (2017). Prevalence of antimicrobial resistant *Escherichia coli* from patients with suspected urinary tract infection in primary care, Denmark. BMC Infect Dis.

[49] Kibret M, Abera B (2011). Antimicrobial susceptibility patterns of *E. coli* from clinical sources in northeast Ethiopia. Afr Health Sci.

[50] Wang Y, Tang C, Yu X, Xia M, Yue H (2010). Distribution of serotypes and virulence-associated genes in pathogenic *Escherichia coli* isolated from ducks. Avian Pathol.

[51] Abd El Tawab AA, Ammar AM, El-Hofy FI, Abdel Hakeem M, Abdel Galil NM (2016). Preliminary studies on *E. coli* implicated in avian colibacillosis with reference to their antibiotic resistance profiles. Banha Vet Med J.

[52] Byomi A, Zidan S, Diab M, Reddy G, Adesiyun A, Abdela W (2017). Characterization of diarrheagenic *Escherichia coli* serotypes isolated from poultry and humans. SOJ Vet Sci.

[53] El-Shabrawy Y, Tarabees R, Hussien AE, Awad A (2019). Bacteriological and molecular studies on virulence encoding genes in *Escherichia coli* isolated from diseased ducks. JCVR..

[54] Enany ME, Algammal AM, Nasef SA, Abo-Eillil SAM, Bin-Jumah M, Taha AE, Allam AA (2019). The occurrence of the multidrug resistance (MDR) and the prevalence of virulence genes and QACs resistance genes in *E. coli* isolated from environmental and avian sources. AMB Express.

[55] Abd El-Mongy M, Abd-El-Moneam GM, Moawad AA, Mohammed AAB (2018). Serotyping and virulence genes detection in *Escherichia coli* isolated from broiler chickens. J Biol Sci.

[56] Saad MS, Hassan MA, Abou El-Roos-Nahla A, Gaafar MH (2017). *E. coli* strains producing Shiga toxin in cattle carcasses at abattoir level. Banha Vet Med J.

[57] Aref NM, Abdel-Raheem AA, Kamaly HF, Hussien SZ (2018). Clinical and sero-molecular characterization of *Escherichia coli* with an emphasis on hybrid strain in healthy and diarrheic neonatal calves in Egypt. Open Vet J.

[58] Murinda SE, Ibekwe AM, Rodriguez NG, Quiroz KL, Mujica AP, Osmon K (2019). Shiga toxin-producing Escherichia coli in mastitis: An international perspective. Foodborne Pathog Dis.

[59] Abe CM, Salvador FA, Falsetti IN, Vieira MA, Blanco J, Blanco JE, Blanco M, Machado AM, Elias WP, Hernandes RT, Gomes TA (2005). Uropathogenic *Escherichia coli* (UPEC) strains may carry virulence properties of diarrhoeagenic *E. coli*. FEMS Immunol Med Microbiol.

[60] World Health Organization: Zoonotic non-O157 Shiga toxin-producing *Escherichia Coli* (STEC). Report of a WHO Scientific Working Group Meeting, Berlin, Germany,1998, 23–26. Available online: [http://whqlibdoc.who.int/hq/1998/WHO_CSR_APH_98.8.pdf]June.

[61] Nissim-Eliraz E, Nir E, Shoval I, Marsiano N, Nissan I, Shemesh H, Nagy N, Goldstein AM, Gutnick M, Rosenshine I, Yagel S, Shpigel NY (2017). Type three secretion system-dependent microvascular thrombosis and ischemic enteritis in human gut xenografts infected with enteropathogenic *Escherichia coli*. Infect Immun.

[62] Ewing W H. Edwards & Ewing identification of *Enterobacteriaceae*, 4th ed. Elsevier Science Publishing. New York, N.Y. 1986.

[63] Levine MM (1987). *Escherichia coli* that cause diarrhoea: enterotoxigenic, enteropathogenic, enteroinvasive, enterohaemorrhagic and enteroadherent. J Infect Dis.

[64] Elsyaed MSAE, Mounir M (2020). Virulence factors and antimicrobial resistance patterns of non-o157 Shiga toxin-producing *Escherichia coli* isolated from different sources at Sadat city. MRJI..

[65] Ranjbar R, Safarpoor Dehkordi F, Sakhaei Shahreza MH, Rahimi E (2018). Prevalence, identification of virulence factors, O-serogroups and antibiotic resistance properties of Shiga-toxin producing *Escherichia coli* strains isolated from raw milk and traditional dairy products. Antimicrob Resist Infect Control.

[66] Kumar A, Taneja N, Kumar Y, Sharma M (2012). Detection of Shiga toxin variants among Shiga toxin-forming Escherichia coli isolates from animal stool, meat and human stool samples in India. J Appl Microbiol.

[67] Koutsoumanis K, Allende A, Alvarez-Ordoenez A, Bover-Cid S, Chemaly M, Davies R, De Cesare A, Herman L, Hilbert F, Lindqvist R, Nauta M, Peixe L, Ru G, Simmons M, Skandamis P, Suffredini E, Jenkins C, Monteiro Pires S, Morabito S, Niskanen T, Scheutz F, da Silva Felicio MT, Messens W, Bolton D, EFSA BIOHAZ Panel (2020). Scientific Opinion on the pathogenicity assessment of Shiga toxin-producing *Escherichia coli* (STEC) and the public health risk posed by contamination of food with STEC. EFSA J.

[68] Farooq S, Hussain I, Mir MA, Bhat MA, Wani SA (2009). Isolation of atypical enteropathogenic Escherichia coli and Shiga toxin 1 and 2f-producing *Escherichia coli* from avian species in India. Lett Appl Microbiol.

[69] Cergole-Novella MC, Nishimura LS, Irino K, Vaz TM, de Castro AF, Leomil L, Guth BE (2006). Stx genotypes and antimicrobial resistance profiles of Shiga toxin-producing Escherichia coli strains isolated from human infections, cattle and foods in Brazil. FEMS Microbiol Lett.

[70] Shi Q, Zhang Y, Gao G, Gao G, Liu Y, Fang H, Chen C, Shen Q (2012). PCR detection of virulence genes Colv, Stxs and HlyE of *Escherichia coli*. J Agric Sci Technol.

[71] Martin A, Beutin L (2011). Characteristics of Shiga toxin-producing Escherichia coli from meat and milk products of different origins and association with food producing animals as main contamination sources. Int J Food Microbiol.

[72] Bielaszewska M, Friedrich AW, Aldick T, Schürk-Bulgrin R, Karch H (2006). Shiga toxin activatable by intestinal mucus in Escherichia coli isolated from humans: predictor for a severe clinical outcome. Clin Infect Dis.

[73] World Health Organization. Critically important antimicrobials for human medicine: ranking of antimicrobial agents for risk management of antimicrobial resistance due to non-human use, 5th rev. Geneva: World Health Organization; Licence: CC BY-NC-SA3.0 IGO (2017). Available online: https://apps.who.int/iris/bitstream/handle/10665/255027/9789241512220-eng.pdf?

[74] Rubab M, Oh DH (2020). Virulence characteristics and antibiotic resistance profiles of Shiga toxin-producing *Escherichia coli* isolates from diverse sources. Antibiotics (Basel).

[75] Krumperman PH (1983). Multiple antibiotic resistance indexing of Escherichia coli to identify high-risk sources of fecal contamination of foods. Appl Environ Microbiol.

[76] Kolár M, Urbánek K, Látal T (2001). Antibiotic selective pressure and development of bacterial resistance. Int J Antimicrob Agents.

[77] Mukherjee S, Mosci RE, Anderson CM, Snyder BA, Collins J, Rudrik JT, Manning SD (2017). Antimicrobial drug-resistant Shiga toxin-producing escherichia coli infections, Michigan, USA. Emerg Infect Dis.

[78] Al-Gendy HA, Hasanen FS, Salem AM, Nada SM (2014). Assessment of oxytetracycline and ampicillin residues in sheep carcasses. Banha Vet Med J.

[79] Okocha RC, Olatoye IO, Adedeji OB (2018). Food safety impacts of antimicrobial use and their residues in aquaculture. Public Health Rev.

[80] Paterson DL, Bonomo RA (2005). Extended-spectrum beta-lactamases: a clinical update. Clin Microbiol Rev.

[81] Kim YB, Yoon MY, Ha JS, Seo KW, Noh EB, Son SH, Lee YJ (2020). Molecular characterization of avian pathogenic Escherichia coli from broiler chickens with colibacillosis. Poult Sci.

[82] Banerjee A, Acharyya S (2020). Molecular characterization of STEC isolated from ducks and its relation to ESBL production. Ukr J Vet Agric Sci.

[83] Kennedy CA, Fanning S, Karczmarczyk M, Byrne B, Monaghan Á, Bolton D, Sweeney T (2017). Characterizing the multidrug resistance of non-O157 Shiga toxin-producing escherichia coli isolates from cattle farms and abattoirs. Microb Drug Resist.

[84] Ahmed AS, Diab HM, Alkahtani MA, Alshehri MA, Saber H, Badr H, et al. Molecular epidemiology of virulent *E. coli* among rural small scale dairy herds and shops: Efficacy of selected marine algal extracts and disinfectants. Int J Environ Health Res. 2020:1–23. 10.1080/09603123.2020.1727422. 10.1080/09603123.2020.1727422 32053006

[85] Saad DN, Sultan S, Abdelhalem MA, Al-Azeem MWA (2019). Molecular detection of blaTEM, blaSHV and blaOXA from *Escherichia coli* isolated from chickens. J Vet Ani Res.

[86] Roberts MC (1996). Tetracycline resistance determinants: mechanisms of action, regulation of expression, genetic mobility, and distribution. FEMS Microbiol Rev.

[87] Sum PE, Sum FW, Projan SJ (1998). Recent developments in tetracycline antibiotics. Curr Pharm Des.

[88] Bryan A, Shapir N, Sadowsky MJ (2004). Frequency and distribution of tetracycline resistance genes in genetically diverse, nonselected, and nonclinical *Escherichia coli* strains isolated from diverse human and animal sources. Appl Environ Microbiol.

[89] Caméléna F, Birgy A, Smail Y, Courroux C, Mariani-Kurkdjian P, Le Hello S, Bonacorsi S, Bidet P (2019). Rapid and simple universal *Escherichia coli* genotyping method based on multiple-locus variable-number tandem-repeat analysis using single-tube multiplex PCR and standard gel electrophoresis. Appl Environ Microbiol.

