# Supplementary material for: Genetic and antimicrobial resistance profiles of non-O157 Shiga toxin-producing Escherichia coli from different sources in Egypt
Source: BMC Microbiol. 2021 Sep 23;21:257. doi: 10.1186/s12866-021-02308-w (PMC8461963; doi:10.1186/s12866-021-02308-w)
Supplement: Supplementary file 1 — Additional file 1 S1. Results of distribution patterns of virulence genes among various E. coli strains. S2. Antimicrobial susceptibility patterns of 60 E. coli strains from different sources. S3. Results of multiple antimicrobial resistance indices of various strains from different sources. S4. Results of distribution of class 1 and 2 integrons, extended-spectrum β-lactamase, and ampicillin-resistance genes in various strains. S5. Results of the distribution patterns of oxytetracycline resistance genes among the phenotypically resistant strains. S6. Results of the gained tandem repeats with the utilized MLVA loci and the discriminatory index. S7. Results of genotypes and allelic profiles of various STEC strains after MLVA typing. S8. Evaluation of the discriminatory power of different MLVA loci combinations. [file 12866_2021_2308_MOESM1_ESM.docx]

**S1.** Results of distribution patterns of virulence genes among various *E. coli* strains.

|  | Gene positive | | | | Virulence genes |
| --- | --- | --- | --- | --- | --- |
| Human ear discharge  (9) | Human urine  (19) | Cattle  (11) | Duck  (14) | Broilers  (7) |  |
| 9/9 (100%) | 19/19 (100%) | 11/11 (100%) | 14/14 (100%) | 7/7 (100%) | *stx*_1_ |
| 8/9 (88.9%) | 14/19 (73.7%) | 8/11 (72.7%) | 14/14 (100%) | 7/7 (100%) | *stx*_1d_ |
| 0/9 (0.0%) | 0/19 (0.0%) | 0/11 (0.0%) | 0/14 (0.0%) | 0/7 (0.0%) | *stx*_1c_ |
| 7/9 (77.8%) | 15/19 (78.9%) | 9/11 (81.8%) | 10/14 (71.4%) | 7/7 (100%) | *stx*_2_ |
| 0/9 (0.0%) | 0/19 (0.0%) | 0/11 (0.0%) | 0/14 (0.0%) | 0/7 (0.0%) | *stx*_2a_ |
| 0/9 (0.0%) | 1/19 (5.3%) | 1/11 (9.1%) | 1/14 (7.1%) | 0/7 (0.0%) | *stx*_2c_ |
| 0/9 (0.0%) | 0/19 (0.0%) | 0/11 (0.0%) | 0/14 (0.0%) | 0/7 (0.0%) | *stx*_2d_ |
| 6/9 (66.7%) | 12/19 (63.4%) | 6/11 (54.5%) | 13/14 (92.9%) | 6/7 (85.7%) | *stx*_2e_ |
| 0/9 (0.0%) | 0/19 (0.0%) | 0/11 (0.0%) | 0/14 (0.0%) | 0/7 (0.0%) | *stx*_2f_ |
| 0/9 (0.0%) | 0/19 (0.0%) | 0/11 (0.0%) | 0/14 (0.0%) | 0/7 (0.0%) | *stx*_2g_ |
| 0/9 (0.0%) | 2/19 (10.5%) | 0/11 (0.0%) | 0/14 (0.0%) | 0/7 (0.0%) | *eae*A |
| 7/9 (77.8%) | 18/19 (94.7%) | 7/11(63.6%) | 8/14 (57.1%) | 6/7 (85.7%) | *ehx*A |

**S2.** Antimicrobial susceptibility patterns of 60 *E. coli* strains from different sources.

| Resistant | | Sensitive | | Antimicrobial agents |
| --- | --- | --- | --- | --- |
| % | No. | % | No. |  |
| 5 | 3 | 95 | 57 | Amikacin |
| 11.7 | 7 | 88.3 | 53 | Amoxicillin/ clavulanic acid |
| 26.7 | 16 | 73.3 | 44 | Ampicillin |
| 65 | 39 | 35 | 21 | Cephradine |
| 50 | 30 | 50 | 30 | Chloramphenicol |
| 81.7 | 49 | 18.3 | 11 | Clindamycin |
| 16.7 | 10 | 83.3 | 50 | Doxycycline |
| 73.3 | 44 | 26.7 | 16 | Erythromycin |
| 41.7 | 25 | 58.3 | 35 | Nalidixic acid |
| 75 | 45 | 25 | 15 | Norociliin |
| 83.3 | 50 | 16.7 | 10 | Oxytetracyclin |
| 96.7 | 58 | 3.9 | 2 | Penicillin G |
| 91.7 | 55 | 8.3 | 5 | Streptomycin |

**S3.** Results of multiple antimicrobial resistance indices of various strains from different sources.

| No | Strains | Origin | Amikacin | Amoxicillin/ clavulanic acid | Ampicillin | Cephradine | Chloramphenicol | Clindamycin | Doxycycline | Erythromycin | Nalidixic acid | Norocillin | Oxytetracycline | Penicillin G | Streptomycin | MAR Index |  |
| --- | --- | --- | --- | --- | --- | --- | --- | --- | --- | --- | --- | --- | --- | --- | --- | --- | --- |
|  |  |  |  |  |  |  |  |  |  |  |  |  |  |  |  |  |  |
| 1 | | O146:H21 | Broiler | S | S | S | R | R | R | S | R | S | R | R | R | R | 0.62 |
| 2 | | O1:H7 | Broiler | S | S | R | R | R | R | S | R | R | R | S | R | R | 0.69 |
| 3 | | O1:H7 | Broiler | S | S | S | S | S | S | S | R | S | R | R | R | R | 0.38 |
| 4 | | O127:H6 | Broiler | S | S | S | R | R | R | S | S | R | S | S | R | R | 0.46 |
| 5 | | O78 | Broiler | S | S | S | R | R | R | R | R | S | R | S | R | R | 0.62 |
| 6 | | O2:H6 | Broiler | S | S | S | R | R | R | S | R | S | R | S | R | R | 0.54 |
| 7 | | O78 | Broiler | S | S | R | R | R | R | S | R | R | R | R | R | R | 0.77 |
| 8 | | O91:H21 | Duck | S | S | S | S | R | R | S | R | S | R | R | R | R | 0.54 |
| 9 | | O78 | Duck | S | S | S | S | S | R | S | S | S | S | R | R | R | 0.31 |
| 10 | | O153:H2 | Duck | S | S | S | S | S | S | S | S | S | R | S | R | R | 0.23 |
| 11 | | O91:H21 | Duck | S | S | S | R | R | R | S | S | S | S | R | R | R | 0.46 |
| 12 | | O2:H6 | Duck | S | S | R | R | R | R | R | R | R | R | R | R | R | 0.85 |
| 13 | | O128:H2 | Duck | S | S | S | R | S | R | S | R | S | R | R | R | R | 0.54 |
| 14 | | O78 | Duck | S | S | S | R | S | R | S | R | S | R | R | R | R | 0.54 |
| 15 | | O2:H6 | Duck | S | S | S | S | R | S | S | R | S | R | R | R | R | 0.46 |
| 16 | | O78 | Duck | S | S | S | R | R | R | R | S | R | S | S | R | R | 0.54 |
| 17 | | O78 | Duck | R | S | R | R | R | R | R | R | R | R | R | R | R | 0.92 |
| 18 | | O26:H11 | Duck | S | S | S | R | S | R | S | R | S | R | S | R | R | 0.46 |
| 19 | | O91:21 | Duck | S | S | S | S | R | S | S | R | S | R | R | R | R | 0.46 |
| 20 | | O128:H2 | Duck | S | S | S | R | R | R | S | S | S | S | R | R | R | 0.46 |
| 21 | | O121:H7 | Duck | S | S | S | R | S | R | S | R | S | R | R | R | R | 0.54 |
| 22 | | O86 | Meat | S | S | S | S | S | R | S | S | S | R | R | R | R | 0.83 |
| 23 | | O111:H2 | Meat | S | S | S | R | R | R | S | R | R | R | R | R | R | 0.69 |
| 24 | | O111:H2 | Meat | S | S | R | R | R | R | S | R | R | R | R | R | R | 0.77 |
| 25 | | O128:H2 | Meat | S | R | R | R | R | R | R | R | R | R | S | R | R | 0.85 |
| 26 | | O26:H11 | Meat | S | S | R | R | R | R | R | R | R | R | S | R | R | 0.77 |
| 27 | | O55:H7 | Milk | S | S | R | S | R | R | S | R | R | R | S | R | R | 0.62 |
| 28 | | O26:H11 | Milk | S | S | S | R | S | R | S | S | S | R | R | R | R | 0.46 |
| 29 | | O55:H7 | Milk | S | S | S | S | S | R | S | R | S | R | R | R | R | 0.46 |
| 30 | | O91:H21 | Milk | R | R | R | R | S | R | R | R | S | R | R | R | R | 0.92 |
| 31 | | O91:H21 | Milk | S | S | S | R | R | R | S | R | S | R | R | R | R | 0.62 |
| 32 | | O127:H6 | Milk | S | S | S | S | S | R | S | R | S | R | R | S | R | 0.38 |
| 33 | | O15:H2 | Human urine | R | R | R | R | R | R | R | S | R | S | R | R | R | 0.85 |
| 34 | | O15:H2 | Human urine | S | S | R | R | R | R | S | R | R | R | R | R | R | 0.77 |
| 35 | | O15:H2 | Human urine | S | S | S | R | S | R | S | R | R | R | R | R | R | 0.62 |
| 36 | | O15:H2 | Human urine | S | S | S | S | R | R | S | R | S | R | R | R | R | 0.54 |
| 37 | | O15:H2 | Human urine | S | S | S | S | S | S | S | R | R | R | R | S | R | 0.38 |
| 38 | | O17:H18 | Human urine | S | R | R | R | R | R | R | S | R | S | R | R | R | 0.85 |
| 39 | | O17:H18 | Human urine | S | S | S | R | S | R | S | R | S | R | R | R | R | 0.54 |
| 40 | | O17:H18 | Human urine | S | R | S | S | S | R | S | R | R | R | R | R | R | 0.62 |
| 41 | | O7:H2 | Human urine | S | S | R | R | R | R | S | R | R | S | R | R | R | 0.69 |
| 42 | | O7:H2 | Human urine | S | S | S | S | S | R | S | R | S | R | R | R | R | 0.46 |
| 43 | | O7:H2 | Human urine | S | S | S | S | S | S | S | R | S | R | R | R | S | 0.31 |
| 44 | | O2:H6 | Human urine | S | R | R | R | R | R | S | S | R | S | R | R | R | 0.69 |
| 45 | | O2:H6 | Human urine | S | S | S | S | S | R | S | R | S | R | R | R | R | 0.46 |
| 46 | | O8:H21 | Human urine | S | S | S | R | S | R | S | R | S | S | R | R | S | 0.38 |
| 47 | | O8:H21 | Human urine | S | S | S | S | S | S | S | R | S | R | R | R | R | 0.38 |
| 48 | | O83 | Human urine | S | S | S | R | S | R | S | S | R | R | R | R | R | 0.54 |
| 49 | | O125:H21 | Human urine | S | S | S | R | S | R | S | R | R | S | R | R | R | 0.54 |
| 50 | | O75 | Human urine | S | S | S | R | S | S | S | R | S | R | R | R | S | 0.38 |
| 51 | | O124 | Human urine | S | S | S | S | S | S | S | S | S | S | R | R | S | 0.15 |
| 52 | | O8:H21 | Human ear discharge | S | S | S | S | S | S | S | R | R | S | R | R | S | 0.31 |
| 53 | | O8:H21 | Human ear discharge | S | S | S | R | S | S | S | R | R | S | R | R | R | 0.46 |
| 54 | | O8:H21 | Human ear discharge | S | S | S | R | S | R | S | R | S | R | R | R | R | 0.54 |
| 55 | | O15:H2 | Human ear discharge | S | S | S | R | R | R | S | S | S | R | R | R | R | 0.54 |
| 56 | | O15:H2 | Human ear discharge | S | S | S | R | R | R | S | S | S | R | R | R | R | 0.54 |
| 57 | | O15:H2 | Human ear discharge | S | S | S | R | S | R | S | S | S | R | R | R | R | 0.46 |
| 58 | | O17:H18 | Human ear discharge | S | R | R | R | R | R | R | R | R | R | R | R | R | 0.92 |
| 59 | | O17:H18 | Human ear discharge | S | S | R | S | R | R | S | R | R | R | R | R | R | 0.69 |
| 60 | | O17:H18 | Human ear discharge | S | S | S | S | S | R | S | R | S | S | R | R | R | 0.38 |

R: resistant, S: sensitive

**S4.** Results of distribution of class 1 and 2 integrons, extended-spectrum β-lactamase, and ampicillin-resistance genes in various strains.

| No. | Strains | Origin | Ampicillin | Integrase 1 (*int*l1) | Integrase 2 (*int*I2) | *bla*CTX-M | *bla*CTX-M-1 | *bla*TEM |
| --- | --- | --- | --- | --- | --- | --- | --- | --- |
|  |  |  |  |  |  |  |  |  |
| 1 | O146:H21 | Broiler | S | - | - | - | - | - |
| 2 | O1:H7 | Broiler | R | - | - | - | + | + |
| 3 | O1:H7 | Broiler | S | - | - | - | - | - |
| 4 | O127:H6 | Broiler | S | - | - | - | - | - |
| 5 | O78 | Broiler | S | - | - | - | - | - |
| 6 | O2:H6 | Broiler | S | - | - | - | - | - |
| 7 | O78 | Broiler | R | - | - | - | + | + |
| 8 | O91:H21 | Duck | S | - | - | - | - | - |
| 9 | O78 | Duck | S | - | - | - | - | - |
| 10 | O153:H2 | Duck | S | - | - | - | - | - |
| 11 | O91:H21 | Duck | S | - | - | - | - | - |
| 12 | O2:H6 | Duck | R | - | - | - | + | - |
| 13 | O128:H2 | Duck | S | - | - | - | - | - |
| 14 | O78 | Duck | S | - | - | - | - | - |
| 15 | O2:H6 | Duck | S | - | - | - | - | - |
| 16 | O78 | Duck | S | - | - | - | - | - |
| 17 | O78 | Duck | R | - | - | - | + | + |
| 18 | O26:H11 | Duck | S | - | - | - | - | - |
| 19 | O91:21 | Duck | S | - | - | - | - | - |
| 20 | O128:H2 | Duck | S | - | - | - | - | - |
| 21 | O121:H7 | Duck | S | - | - | - | - | - |
| 22 | O86 | Meat | S | - | - | - | - | - |
| 23 | O111:H2 | Meat | S | - | - | - | - | - |
| 24 | O111:H2 | Meat | R | - | - | - | + | - |
| 25 | O128:H2 | Meat | R | - | - | - | + | - |
| 26 | O26:H11 | Meat | R | - | - | - | + | - |
| 27 | O55:H7 | Milk | R | - | - | - | + | - |
| 28 | O26:H11 | Milk | S | - | - | - | - | - |
| 29 | O55:H7 | Milk | S | - | - | - | - | - |
| 30 | O91:H21 | Milk | R | - | - | - | - | - |
| 31 | O91:H21 | Milk | S | - | - | - | - | - |
| 32 | O127:H6 | Milk | S | - | - | - | - | - |
| 33 | O15:H2 | Human urine | R | - | - | - | - | - |
| 34 | O15:H2 | Human urine | R | - | - | - | - | - |
| 35 | O15:H2 | Human urine | S | - | - | - | - | - |
| 36 | O15:H2 | Human urine | S | - | - | - | - | - |
| 37 | O15:H2 | Human urine | S | - | - | - | - | - |
| 38 | O17:H18 | Human urine | R | - | - | - | - | - |
| 39 | O17:H18 | Human urine | S | - | - | - | - | - |
| 40 | O17:H18 | Human urine | S | - | - | - | - | - |
| 41 | O7:H2 | Human urine | R | - | - | - | - | - |
| 42 | O7:H2 | Human urine | S | - | - | - | - | - |
| 43 | O7:H2 | Human urine | S | - | - | - | - | - |
| 44 | O2:H6 | Human urine | R | - | - | - | - | - |
| 45 | O2:H6 | Human urine | S | - | - | - | - | - |
| 46 | O8:H21 | Human urine | S | - | - | - | - | - |
| 47 | O8:H21 | Human urine | S | - | - | - | - | - |
| 48 | O83 | Human urine | S | - | - | - | - | - |
| 49 | O125:H21 | Human urine | S | - | - | - | - | - |
| 50 | O75 | Human urine | S | - | - | - | - | - |
| 51 | O124 | Human urine | S | - | - | - | - | - |
| 52 | O8:H21 | Human ear discharge | S | - | - | - | - | - |
| 53 | O8:H21 | Human ear discharge | S | - | - | - | - | - |
| 54 | O8:H21 | Human ear discharge | S | - | - | - | - | - |
| 55 | O15:H2 | Human ear discharge | S | - | - | - | - | - |
| 56 | O15:H2 | Human ear discharge | S | - | - | - | - | - |
| 57 | O15:H2 | Human ear discharge | S | - | - | - | - | - |
| 58 | O17:H18 | Human ear discharge | R | - | - | - | - | - |
| 59 | O17:H18 | Human ear discharge | R | - | - | - | - | - |
| 60 | O17:H18 | Human ear discharge | S | - | - | - | - | - |
| Total |  |  |  | 0/60 (0.0%) | 0/60 (0.0%) | 0/60 (0.0%) | 8/60 (13.3%) | 3/60 (5%) |

(+) positive, (-) negative

**S5.** Results of the distribution patterns of oxytetracycline resistance genes among the phenotypically resistant strains.

| Code | Strains | Origin | Oxytetracycline | *tet*A | *tet*B | *tet*C | *tet*D | *tet*E | *tet*G | No. Of genes per each strain |
| --- | --- | --- | --- | --- | --- | --- | --- | --- | --- | --- |
| 1 | O 146:H21 | Broiler | R | + | + | - | - | - | + | 3 |
| 2 | O1:H7 | Broiler | S | - | - | - | - | - | - | 0 |
| 3 | O1:H7 | Broiler | R | + | + | - | - | - | + | 3 |
| 4 | O127:H6 | Broiler | S | - | - | - | - | - | - | 0 |
| 5 | O78 | Broiler | S | - | - | - | - | - | - | 0 |
| 6 | O2:H6 | Broiler | S | - | - | - | - | - | - | 0 |
| 7 | O78 | Broiler | R | + | + | + | - | + | + | 5 |
| 8 | O91:H21 | Duck | R | + | + | - | - | + | + | 4 |
| 9 | O78 | Duck | R | + | + | - | - | + | + | 4 |
| 10 | O153:H2 | Duck | S | - | - | - | - | - | - | 0 |
| 11 | O91:H21 | Duck | R | + | + | - | - | + | - | 3 |
| 12 | O2:H6 | Duck | R | + | + | - | - | + | - | 3 |
| 13 | O128:H2 | Duck | R | + | + | + | - | + | + | 5 |
| 14 | O78 | Duck | R | + | + | - | - | + | - | 3 |
| 15 | O2:H6 | Duck | R | + | + | - | - | + | - | 3 |
| 16 | O78 | Duck | S | - | - | - | - | - | - | 0 |
| 17 | O78 | Duck | R | + | + | - | - | + | + | 4 |
| 18 | O26:H11 | Duck | S | - | - | - | - | - | - | 0 |
| 19 | O91:21 | Duck | R | + | + | - | - | + | - | 3 |
| 20 | O128:H2 | Duck | R | + | + | - | - | - | + | 3 |
| 21 | O121:H7 | Duck | R | + | + | - | - | + | - | 3 |
| 22 | O86 | Meat | R | + | + | - | - | + | + | 4 |
| 23 | O111:H2 | Meat | R | + | + | - | - | + | + | 4 |
| 24 | O111:H2 | Meat | R | + | + | - | - | + | + | 4 |
| 25 | O128:H2 | Meat | S | - | - | - | - | - | - | 0 |
| 26 | O26:H11 | Meat | S | - | - | - | - | - | - | 0 |
| 27 | O55:H7 | Milk | S | - | - | - | - | - | - | 0 |
| 28 | O26:H11 | Milk | R | + | + | - | - | + | - | 3 |
| 29 | O55:H7 | Milk | R | + | + | - | - | + | + | 4 |
| 30 | O91:H21 | Milk | R | + | + | - | - | + | + | 4 |
| 31 | O91:H21 | Milk | R | + | + | - | - | + | + | 4 |
| 32 | O127:H6 | Milk | R | + | - | - | - | + | + | 3 |
| 33 | O15:H2 | Human urine | R | + | + | - | - | + | + | 4 |
| 34 | O15:H2 | Human urine | R | + | + | - | - | + | + | 4 |
| 35 | O15:H2 | Human urine | R | - | + | - | - | + | + | 3 |
| 36 | O15:H2 | Human urine | R | - | - | - | - | + | + | 2 |
| 37 | O15:H2 | Human urine | R | + | + | - | - | + | + | 4 |
| 38 | O17:H18 | Human urine | R | + | + | - | - | + | + | 4 |
| 39 | O17:H18 | Human urine | R | - | - | - | - | + | + | 2 |
| 40 | O17:H18 | Human urine | R | + | + | - | - | + | - | 3 |
| 41 | O7:H2 | Human urine | R | - | + | - | - | + | - | 2 |
| 42 | O7:H2 | Human urine | R | - | + | - | - | + | - | 2 |
| 43 | O7:H2 | Human urine | R | + | - | - | - | + | - | 2 |
| 44 | O2:H6 | Human urine | R | - | - | - | - | + | - | 1 |
| 45 | O2:H6 | Human urine | R | - | + | - | - | + | - | 2 |
| 46 | O8:H21 | Human urine | R | + | + | - | - | + | - | 3 |
| 47 | O8:H21 | Human urine | R | + | + | - | - | + | - | 3 |
| 48 | O83 | Human urine | R | + | + | - | - | + | - | 3 |
| 49 | O125:H21 | Human urine | R | + | + | - | - | + | - | 3 |
| 50 | O75 | Human urine | R | - | + | - | - | + | - | 2 |
| 51 | O124 | Human urine | R | + | + | - | - | + | - | 3 |
| 52 | O8:H21 | Human ear discharge | R | - | - | - | - | + | - | 1 |
| 53 | O8:H21 | Human ear discharge | R | + | + | - | - | + | - | 3 |
| 54 | O8:H21 | Human ear discharge | R | + | + | - | - | + | + | 4 |
| 55 | O15:H2 | Human ear discharge | R | + | + | - | - | + | + | 4 |
| 56 | O15:H2 | Human ear discharge | R | + | + | - | - | + | + | 4 |
| 57 | O15:H2 | Human ear discharge | R | + | + | - | - | + | - | 3 |
| 58 | O17:H18 | Human ear discharge | R | + | + | - | - | + | + | 4 |
| 59 | O17:H18 | Human ear discharge | R | + | + | - | - | + | + | 4 |
| 60 | O17:H18 | Human ear discharge | R | - | + | - | - | - | - | 1 |
| Total | | | | 40/60  (66.7%) | 44/60  (73.3%) | 2/60  (3.3%) | 0/60  (0.0 %) | 46/60  (76.7%) | 27/60  (45%) |  |

(+) positive, (-) negative

Percentage %

Shiga toxins and their variants

**Figure 1.** Percentage of virulence genes among different strains from various hosts.

Used antimicrobials

Percentage of sensitive and resistant strains %

**Figure 2.** Efficacy of 13 antimicrobials on the isolated *E. coli* strains.

Percentage of the genotypes %

The prevalent genotypes

**Figure 3.** Percentage of the obtained genotypes after MLVA analysis.

**S6.** Results of the gained tandem repeats with the utilized MLVA loci and the discriminatory index.

| Locus | No. of strains per each (h) calculated with the various numbers of copies | | | | | | | | | | | | | | | | | | | | | | | | | | Hunter–Gaston  discriminatory index  (DI) | 95% CI |
| --- | --- | --- | --- | --- | --- | --- | --- | --- | --- | --- | --- | --- | --- | --- | --- | --- | --- | --- | --- | --- | --- | --- | --- | --- | --- | --- | --- | --- |
|  | 0 | 1 | 2 | 3 | 4 | 5 | 6 | 7 | 8 | 9 | 10 | 11 | 12 | 13 | 14 | 15 | 16 | 17 | 18 | 19 | 20 | 21 | 22 | 23 | 24 | 25 |  |  |
| **VNTR_3** | 4 |  |  |  |  |  |  |  |  |  |  |  | 26 | 27 | 3 |  |  |  |  |  |  |  |  |  |  |  | 0.613 | 1.000 (1.000**–**1.000) |
| **VNTR_34** |  |  |  |  | 52 | 8 |  |  |  |  |  |  |  |  |  |  |  |  |  |  |  |  |  |  |  |  | 0.235 | 1.000 (1.000**–**1.000) |
| **VNTR_9** |  | 44 |  |  |  |  |  |  |  |  |  |  |  |  |  |  |  |  |  |  |  |  |  |  |  | 16 | 0.3977 | 1.000 (1.000**–**1.000) |
| **VNTR_25** |  | 4 |  |  |  |  |  | 53 |  |  |  |  |  | 3 |  |  |  |  |  |  |  |  |  |  |  |  | 0.2164 | 1.000 (1.000**–**1.000) |
| **VNTR_17** |  |  |  |  |  |  |  |  |  |  |  |  |  |  |  | 27 |  |  |  |  |  | 33 |  |  |  |  | 0.5034 | 1.000 (1.000**–**1.000) |
| **VNTR_19** |  |  | 36 |  |  | 24 |  |  |  |  |  |  |  |  |  |  |  |  |  |  |  |  |  |  |  |  | 0.4881 | 1.000 (1.000**–**1.000) |
| **VNTR_36** |  |  |  |  |  | 30 |  | 1 |  |  |  |  |  |  |  |  |  |  |  |  | 29 |  |  |  |  |  | 0.5249 | 1.000 (1.000**–**1.000) |
| **VNTR_37** |  |  |  | 12 |  |  |  |  |  |  |  |  |  |  |  |  |  |  |  |  |  |  | 48 |  |  |  | 0.3254 | 1.000 (1.000**–**1.000) |

**S 7.** Results of genotypes and allelic profiles of various STEC strains after MLVA typing.

| Genotype | Allelic profile | | | | | | | | Strains,  hosts, and codes | Percentage |
| --- | --- | --- | --- | --- | --- | --- | --- | --- | --- | --- |
| 1 | 13 | 4 | 25 | 13 | 21 | 5 | 20 | 22 | O146:H21 (broiler 1) | 1/60 (1.7%) |
| 2 | 13 | 5 | 1 | 7 | 21 | 5 | 20 | 22 | O1:H7 (broiler 2), O2:H6 (broiler 6), O78 (broiler 7), O153:H2 (duck 10), O91:H21 (duck 11) | 5/60 (8.3%) |
| 3 | 12 | 4 | 25 | 7 | 21 | 5 | 20 | 22 | O1:H7 (broiler3), O127:H6 (broiler 4), O78 (duck 9) | 3/60 (5%) |
| 4 | 12 | 5 | 1 | 7 | 21 | 5 | 20 | 22 | O78 (broiler 5), O91:H21 (duck 8) | 2/60 (3.3%) |
| 5 | 13 | 4 | 1 | 1 | 21 | 5 | 20 | 22 | O2:H6 (duck 12) | 1/50 (1.7%) |
| 6 | 13 | 4 | 25 | 7 | 21 | 5 | 20 | 22 | O128:H2 (duck 13), O78 (duck 14), O2:H6 (duck 15), O26:H11 (duck 18), O111:H2 (beef 24) | 5/60 (8.3%) |
| 7 | 13 | 4 | 25 | 1 | 21 | 5 | 20 | 22 | O78 (duck 16) | 1/60 (1.7%) |
| 8 | 13 | 5 | 1 | 1 | 21 | 5 | 20 | 22 | O78 (duck 17) | 1/60 (1.7%) |
| 9 | 13 | 4 | 25 | 7 | 21 | 2 | 20 | 22 | O91:21 (duck 19) | 1/60 (1.7%) |
| 10 | 13 | 4 | 1 | 7 | 21 | 5 | 20 | 22 | O128:H2 (duck 20), O111:H2 (beef 23) | 2/60 (3.3%) |
| 11 | 13 | 4 | 1 | 7 | 21 | 2 | 20 | 22 | O121:H7 (duck 21), O86 (beef 22) | 2/60 (3.3%) |
| 12 | 14 | 4 | 25 | 7 | 21 | 5 | 20 | 22 | O128:H2 (beef 25) | 1/60 (1.7%) |
| 13 | 14 | 4 | 1 | 7 | 21 | 2 | 20 | 22 | O26:H11 (beef 26) | 1/60 (1.7%) |
| 14 | 14 | 4 | 25 | 7 | 21 | 2 | 20 | 22 | O55:H7 (cow’s milk 27) | 1/60 (1.7%) |
| 15 | 13 | 4 | 25 | 7 | 15 | 2 | 20 | 22 | O26:H11 (cow’s milk 28) | 1/60 (1.7%) |
| 16 | 13 | 4 | 1 | 13 | 21 | 2 | 20 | 3 | O55:H7 (cow’s milk 29) | 1/60 (1.7%) |
| 17 | 13 | 4 | 25 | 7 | 15 | 2 | 7 | 3 | O91:H21 (cow’s milk 30) | 1/60 (1.7%) |
| 18 | 13 | 4 | 1 | 7 | 21 | 2 | 5 | 3 | O127:H6 (cow’s milk 32) | 1/60 (1.7%) |
| 19 | 13 | 4 | 1 | 7 | 21 | 2 | 5 | 22 | O91:H21 (cow’s milk 31), O15:H2 (human 33), O15:H2 (human 34), O15:H2 (human 35) | 4/60 (6.7 %) |
| 20 | 12 | 4 | 1 | 7 | 15 | 5 | 5 | 22 | O15:H2 (human 36), O7:H2 (human 42) | 2/60 (3.3%) |
| 21 | 12 | 4 | 1 | 7 | 15 | 2 | 5 | 22 | O15:H2 (human 37), O17:H18 (human 40), O7:H2 (human 40), O7:H2 (human 43), O8:H21 (human 46), O8:H21 (human 47), O8:H21 (human52), O8:H21 (human 53), O8:H21 (human 54), O15:H2 (human 55), O15:H2 (human 56), O15:H2 (human 57) | 12/60 (20 %) |
| 22 | 12 | 4 | 1 | 7 | 15 | 2 | 5 | 3 | O17:H18 (human 38), O83 (human 48), O125:H21 (human 49), O17:H18 (human 58), O17:H18 (human 59),  O17:H18 (human 60) | 6/60 (10%) |
| 23 | 0 | 4 | 1 | 1 | 15 | 2 | 5 | 3 | O17:H18 (human 39) | 1/60 (1.7%) |
| 24 | 0 | 4 | 1 | 7 | 15 | 2 | 5 | 3 | O2:H6 (human 44), O2:H6 (human 45) | 2/60 (3.3%) |
| 25 | 0 | 4 | 1 | 7 | 15 | 2 | 5 | 22 | O75 (human 50) | 1/60 (1.7%) |
| 26 | 12 | 4 | 25 | 13 | 15 | 2 | 5 | 22 | O124 (human 51) | 1/60 (1.7%) |

**S8.** Evaluation of the discriminatory power of different MLVA loci combinations.

| Loci combinations | No. of  clusters | No. of isolates  in an individual  cluster | DI | 95% CI |
| --- | --- | --- | --- | --- |
| VNTR_3, VNTR_34,  VNTR_9, VNTR_25,  VNTR_17,VNTR_19, VNTR_36, VNTR_37 | 23 | 1-12 | 0.9277 | 1.000 (1.000**–**1.000) |
| VNTR_3,VNTR_9,  VNTR_17,VNTR_19,  VNTR_36, VNTR_37 | 18 | 1-12 | 0.9138 | 1.000 (1.000**–**1.000) |
| VNTR_25, VNTR_34 | 5 | 1-46 | 0.6161 | 1.000 (1.000**–**1.000) |
